# Mobile genetic elements carrying aminoglycoside resistance genes in *Acinetobacter baumannii* isolates belonging to global clone 2

**DOI:** 10.3389/fmicb.2023.1172861

**Published:** 2023-05-05

**Authors:** Ghazal Naderi, Malihe Talebi, Roghayeh Gheybizadeh, Arash Seifi, Sedigheh Ghourchian, Mohammad Rahbar, Alireza Abdollahi, Abdolhossein Naseri, Parisa Eslami, Masoumeh Douraghi

**Affiliations:** ^1^Division of Microbiology, Department of Pathobiology, School of Public Health, Tehran University of Medical Sciences, Tehran, Iran; ^2^Department of Microbiology, School of Medicine, Iran University of Medical Sciences, Tehran, Iran; ^3^Department of Infectious Diseases, Faculty of Medicine, Tehran University of Medical Sciences, Tehran, Iran; ^4^Department of Microbiology, Iranian Reference Health Laboratory Research Center, Ministry of Health and Medical Education, Tehran, Iran; ^5^Department of Pathology, Imam Hospital Complex, Tehran University of Medical Sciences Tehran, Iran; ^6^Department of Laboratory Sciences, School of Paramedical Sciences, Iran University of Medical Sciences, Tehran, Iran; ^7^Department of Microbiology, Milad Hospital, Tehran, Iran

**Keywords:** *Acinetobacter baumannii* genomic resistance island, aminoglycoside resistance, global clone 2, mobile genetic element (MGE), plasmids

## Abstract

Aminoglycosides are used to treat infections caused by carbapenem-resistant *Acinetobacter baumannii* (CRAB) strains. However, resistance to aminoglycosides has increased remarkably in the last few years. Here, we aimed to determine the mobile genetic elements (MGEs) associated with resistance to aminoglycosides in the global clone 2 (GC2) *A. baumannii*. Among the 315 *A. baumannii* isolates, 97 isolates were identified as GC2, and 52 of GC2 isolates (53.6%) were resistant to all the aminoglycosides tested. The AbGRI3s carrying *armA* were detected in 88 GC2 isolates (90.7%), and of them, 17 isolates (19.3%) carried a new variant of AbGRI3 (AbGRI3_ABI221_). *aphA6* was located in Tn*aphA6* of 30 isolates out of 55 *aphA6*-harboring isolates, and 20 isolates were found to harbor Tn*aphA6* on a RepAci6 plasmid. Tn*6020* carrying *aphA1b* was detected in 51 isolates (52.5%), which was located within AbGRI2 resistance islands. The pRAY^*^ carrying the *aadB* gene was detected in 43 isolates (44.3%), and no isolate was found to contain a class 1 integron harboring this gene. The GC2 *A. baumannii* isolates contained at least one MGE carrying the aminoglycoside resistance gene, located mostly either in the chromosome within AbGRIs or on the plasmids. Thus, it is likely that these MGEs play a role in the dissemination of aminoglycoside resistance genes in GC2 isolates from Iran.

## Introduction

*Acinetobacter baumannii* causes a variety of healthcare-associated infections (HAIs), which are mostly untreatable by currently available antibiotics. Most of these antibiotics are ineffective against *A. baumannii* as it has a high capacity to acquire antibiotic-resistant genes (ARGs) via mobile genetic elements (MGE), including plasmids, transposons, integrons, and resistance islands (Fournier and Richet, 2006; Towner, 2009). The majority of *A. baumannii* isolates that are resistant to multiple antibiotics belong to one of two major global clones, namely, global clone 1 (GC1) and global clone 2 (GC2) (Holt et al., 2016). Carbapenem-resistant *A. baumannii* (CRAB) strains are among the critical priority pathogens on the World Health Organization's (WHO's) priority list of antibiotic-resistant bacteria (Tacconelli et al., 2018). Aminoglycosides are used as antibiotics of choice in the treatment of infections caused by CRAB strains; however, resistance to antibiotics of this class is prominently increased in recent years (Nemec et al., 2004; Lee et al., 2011).

The MGEs carrying aminoglycoside resistance genes have been identified in *A. baumannii* isolates belonging to GC1 and GC2 (Nigro et al., 2011, 2013; Hamidian et al., 2012; Nigro and Hall, 2016; Blackwell et al., 2017; Hamidian and Hall, 2018). In GC2 *A. baumannii*, the chromosomally located *A. baumannii* genomic resistance islands (AbGRIs) often carry aminoglycoside resistance genes, including *strA, strB, aphA1b, aacC1, aadA1, armA*, and *aacA4* (Nigro et al., 2013; Nigro and Hall, 2016; Blackwell et al., 2017). The AbGRI1 resistance islands containing Tn*6022* and/or Tn*6022*Δ*1* backbone transposons, which are located in the *comM* gene, carry *strA* and *strB* genes (streptomycin resistance) (Nigro and Hall, 2016). In some of the AbGRI2 variants, *aacC1* (gentamicin resistance), *aadA1* (streptomycin and spectinomycin resistance), and *aphA1b* (kanamycin and neomycin resistance) genes are co-located. Tn*6020* (a compound transposon flanked by IS26) that carries the *aphA1b* gene (conferring resistance to kanamycin and neomycin) has been found in some of the *AbGRI2* and *AbGRI3* variants (Nigro et al., 2013; Nigro, 2014; Nigro and Hall, 2016; Blackwell, 2017; Blackwell et al., 2017). AbGRI3 resistance islands carry an IS26-bounded transposon (Tn*6180*) containing a 16S rRNA methyltransferase (*armA*) gene, which confers resistance to most of therapeutic aminoglycosides (Galimand et al., 2003; Blackwell et al., 2017). Furthermore, some variants of the AbGRI3 resistance island carry the *aacA4* gene, conferring resistance to amikacin, kanamycin, and neomycin (Blackwell et al., 2017). In addition to the AbGRI resistance islands harboring aminoglycoside resistance genes (Nigro et al., 2013; Nigro and Hall, 2016; Blackwell et al., 2017), plasmids have a significant role in the carriage of genes responsible for aminoglycoside resistance (Nigro et al., 2011, 2015; Hamidian et al., 2012). The *aphA6* gene, which confers resistance to amikacin, kanamycin, and neomycin, is most commonly located within Tn*aphA6* (a transposon bounded by copies of ISAba125), which is frequently found on the RepAci6 plasmid family (Nigro and Hall, 2014; Nigro et al., 2015). The *aadB* gene cassette, which confers resistance to tobramycin, gentamicin, and kanamycin, is usually associated with a class 1 integron or a plasmid named pRAY (Nigro et al., 2011; Hamidian et al., 2012). There are several reports of resistance to aminoglycosides in *A. baumannii* isolates from Iran (Aliakbarzade et al., 2014) and other Middle East countries (Bakour et al., 2014; Kishk et al., 2021; Sannathimmappa et al., 2021; Al-Tamimi et al., 2022); however, little is known regarding the role of MGEs in the dissemination of these resistance genes in the Middle East region (Aris et al., 2019; Douraghi et al., 2020). Here, we examined the aminoglycoside resistance genes and their association with MGEs in GC2 isolates in Tehran, Iran.

## Materials and methods

### Bacterial isolates

A total of 315 non-repetitive *A. baumannii* isolates were recovered from patients admitted to one of the four hospitals located in Tehran, Iran (H1, H2, H4, and H5), in the periods 2012–2013 (*n* = 111) and 2018–2019 (*n* = 204). All isolates were initially identified using conventional microbiological methods (Forbes and Sahm, 2016) and were further confirmed by the detection of the *oxaAb* (*bla*_OXA − 51 − like_) gene by PCR (Turton et al., 2006).

### Antibiotic susceptibility testing

Antibiotic susceptibility testing was performed by the disk diffusion method using the following 27 antibiotics (μg per disk): ampicillin–sulbactam (20), piperacillin–tazobactam (110), ticarcillin–clavulanate (timentin) (85), imipenem (10), meropenem (10), doripenem (10), ceftazidime (30), cefotaxime (30), cefepime (30), ceftriaxone (30), tetracycline (30), minocycline (30), doxycycline (30), streptomycin (25), spectinomycin (25), amikacin (30), kanamycin (30), gentamicin (10), neomycin (30), netilmicin (30), tobramycin (10), nalidixic acid (30), ciprofloxacin (5), levofloxacin (5), sulfamethoxazole (300), trimethoprim–sulfamethoxazole (25), and rifampin (30). The results were analyzed according to the Clinical and Laboratory Standards Institute (CLSI) recommendations for *Acinetobacter* spp. (Wayne, 2022) and calibrated dichotomous sensitivity (CDS) (http://cdstest.net/) disk diffusion assay when a CLSI breakpoint for *Acinetobacter* spp. was not available (CDS for streptomycin, spectinomycin, kanamycin, neomycin, netilmicin, nalidixic acid, sulfamethoxazole, and rifampin).

### PCR assays, DNA sequencing, and sequence analysis

For PCR amplicons up to 3 kb, reaction and cycling conditions were followed as described in Hamidian and Hall (2011). For larger amplicons, Phusion DNA polymerase (New England Biolabs) and Phusion HF buffer were used instead of *Taq* polymerase and PCR buffer. The identity of PCR amplicons was confirmed by DNA sequencing (Nigro et al., 2011). The amplicon of PCR linking the left end of AbGRI3 to ISAba24 (Δ*atr*-ISAba24), which was larger than the predicted size, was sequenced by primer walking strategy (primers are presented in Supplementary Table 1).

### Identification of *A. baumannii* isolates belonging to GC2

The *A. baumannii* isolates belonging to GC2 were identified by two multiplex PCRs, as described previously (Turton et al., 2007).

### Detection of aminoglycoside resistance genes

The following aminoglycoside resistance genes were identified by PCR in GC2 *A. baumannii* isolates, including *strA, strB, aacC1, aacC2, aacA4, aphA1b, aphA6, aadA1, armA, aadB*, and *aac(6')-Im* (Nigro et al., 2011).

### Determining the context of aminoglycoside resistance genes

**Figure 1:**
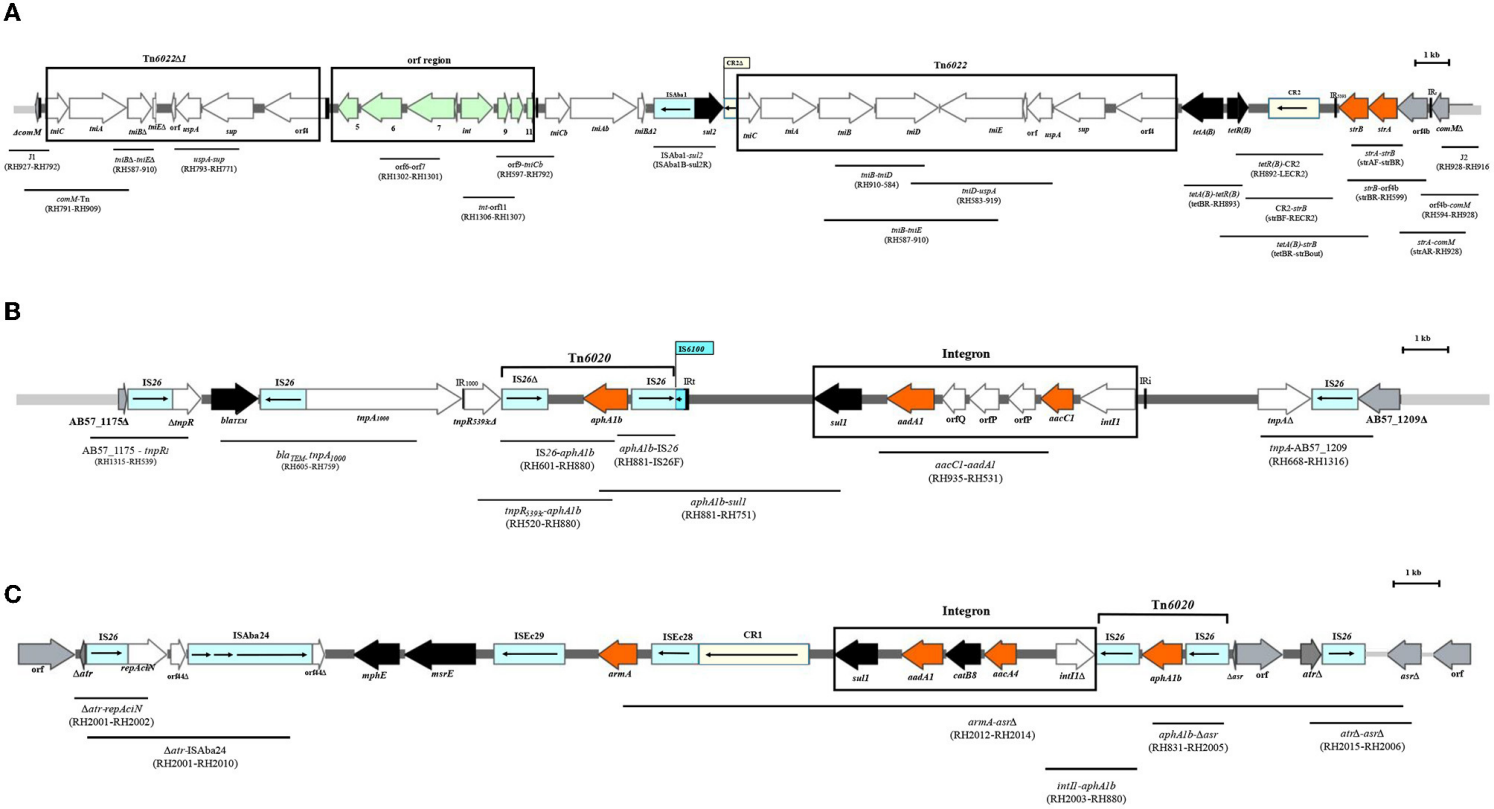
The linkage of PCRs to map the backbone of AbGRI resistance islands. **(A)** Schematic of the AbGRI1-3. **(B)** Schematic of the AbGRI2-1. **(C)** Schematic of the AbGRI3-2i. The central dark gray line represents the resistance island. The light gray line indicates the adjacent chromosomal sequence. Arrows indicate the extent and orientation of genes and open reading frames. The aminoglycoside resistance genes are orange and other resistance genes are black with the name below. The boxes representing IS are blue and the boxes representing CR elements are yellow, and the internal arrows indicate the transposase or the rolling circle replicase, respectively. Vertical bars indicate IRs, and the triangles next to the genes indicate the deletion in the genes. The location of the Tn*6022*, Tn*6022*Δ1, integron, and orf region is shown by the black box around them. The primers used to link sequences are indicated beneath each black line in parentheses. Figure made adapted from Blackwell et al. (2017).

The aminoglycoside resistance genes associated with AbGRI resistance islands (Figure 1), including *strA* and *strB* with AbGRI1, *aphA1b* within Tn*6020* with AbGRI2, *aacC1* alongside *aadA1* within the class 1 integron with AbGRI2, and *armA* with AbGRI3 resistance islands were identified by PCR and PCR mapping using the primers presented in Supplementary Table 2. Furthermore, the *aphA6* within Tn*aphA6* associated with RepAci6 and the *aadB* associated with pRAY^*^ or class 1 integron were identified by PCR and PCR mapping using the primers presented in Supplementary Table 2.

### Accession numbers

The partial sequences of the AB57_1175-*tnpR*_1_, *bla*_*TEM*−_ *tnpA*_1000_*, tnpR*_5393*c*_*-aphA1b, tnpA*_21_*-*AB57_1209*, armA* gene, and Δ*atr*-ISAba24 have been submitted to the NCBI GenBank database under the following accession numbers: OM801571, ON240823, ON871819, OP019034, ON982224, and OQ341795.

## Results

### Identification of GC2 *A. baumannii* isolates and antibiotic resistance profiles

Among the 315 *A. baumannii* isolates, 97 (30.8%) belonged to GC2, and all the GC2 isolates were resistant to carbapenems (imipenem, meropenem, and doripenem) (Supplementary Table 3). As each of the GC2 isolates were resistant to at least an antibiotic in the three classes (Magiorakos et al., 2012), they were classified as multi-drug resistant (MDR) (Supplementary Table 3). The aminoglycoside resistance profile of the GC2 isolates varied, and four groups were distinguished in the collection, hereafter will be referred to as groups 1–4 (Table 1). While the isolates in the group 1 were resistant to all aminoglycosides tested, the isolates in the remaining three groups were susceptible to neomycin (group 2), netilmicin (group 3), and both tobramycin and netilmicin (group 4).

**Table 1:** Aminoglycoside resistance profiles in GC2 *A. baumannii* isolates.

**Group**	**Isolates**	**Aminoglycoside resistance genes detected**	**Aminoglycoside resistance phenotype**
1	ABS574, ABM304, ABM313, ABM331, ABM334, ABM379, ABM382, ABM391, ABM395, ABM429	*armA, aphA1b*	Sm Ak Gm Km Ne Nm Tm
	ABM465, ABH019	*armA, aphA1b, strA, strB*	
	ABM440, ABM445, ABH001, ABH006	*armA, aphA6, strA, strB*	
	ABS564, ABS580, ABS583, ABM310, ABM380, ABM402, ABM438	*armA, aphA6, aphA1b*	
	ABM476, ABH065	*armA, aadB, aphA6, strA, strB*	
	ABH013	*armA, aacC1, aphA6, strA, strB*	
	ABM315, ABM368, ABM399, ABM432, ABM435	*armA, aphA1b, aadB, strA, strB*	
	ABS573, ABM393	*armA, aphA1b, aadB*,	
	ABS568	*armA, aacC1, aadB, aphA6*	
	ABS569, ABS570, ABS571, ABS572, ABS575, ABS577, ABS582, ABS588, ABS593, ABS614, ABM305, ABM337, ABM341, ABM366, ABM377	*armA, aphA1b, aadB, aphA6*	
	ABS565	*armA, aadA1, aphA1b, aadB, aphA6, strA, strB*	
2	ABS567, ABS581, ABM444, ABM342, ABM343, ABM463	*armA*	Sm Ak Gm Km Ne Tm
	ABM338, ABM428, ABM430, ABM459, ABM467, ABH014	*armA, strA, strB*	
	ABM323ABM346, ABM316, ABM324	*armA, aadB*	
	ABS534, ABM345, ABM390, ABM473, ABM474, ABM475, ABM329	*armA, aadB, strA, strB*	
	ABM433, ABM434, ABM441, ABM442, ABM460, ABM461, ABM462, ABM466, ABM469, ABM471, ABH003, ABH007	*armA, aphA6, strA, strB*	
	ABM468	*armA, aadA1, aphA6, strA, strB*	
	ABM322	*armA, aadB, aphA6*	
	ABS594	*armA, aadA1, aadB, aphA6, strA, strB*	
3	ABS566, ABM319, ABM392	*aacC1, aphA1b, aadB, aphA6, strA, strB*	Sm Ak Gm Km Nm Tm
	ABM378	*aacC1, aadA1, aphA1b, aadB, aphA6, strA, strB*	
4	ABM336	*aacC1, aphA1b, aphA6*	Sm Ak Gm Km Nm
	ABS470, ABS495, ABS496, ABM472	*aacC1, aphA1b, aphA6, strA, strB*	

Sm, Streptomycin; Ak, Amikacin; Gm, Gentamicin; Km, Kanamycin; Ne, Netilmicin; N, Neomycin; Tm, Tobramycin.

### Detection of aminoglycoside resistance genes

The *armA* was the most frequent gene, detected in all but nine (*n* = 88) of GC2 *A. baumannii* isolates (group 1 and group 2 in Table 1). Furthermore, the *aphA6* (*n* = 55), *aphA1b* (*n* = 51), *aadB* (*n* = 43), *aacC1* (*n* = 11), and *aadA1* (*n* = 4) genes were detected in the GC2 isolates. The *aacC2, aacA4*, and *aac(6')-Im* genes were not observed in any isolate examined (Supplementary Table 4).

### The context of *strA* and *strB* genes

The *strA* and *strB* genes were co-located in all isolates carrying AbGRI1 resistance island (Supplementary Table 5, highlighted). In total, 51 out of the 97 GC2 isolates tested (52.6%) did not contain the AbGRI1 resistance island (Supplementary Table 5, not highlighted). In the remaining 46 isolates (47.4%), seven groups were distinguished according to their shared characteristics (Supplementary Table 5, each group highlighted in the same color). The interrupted *comM* gene; J1, J2 junction; orf4b adjacent to the *comM* gene (indicating that they contained AbGRI1 resistance island); *strA, strB* genes; and CR2 element were present in all isolates containing AbGRI1. However, the isolates carrying AbGRI1 varied according to the presence of backbone transposon (Tn*6022*, Tn*6022*Δ*1*); orf region; *tetA(B), tetR(B)* (tetracycline resistance); and *sul2* gene (sulfonamide resistance) (Supplementary Table 5, highlighted).

Long-read sequencing technology such as PacBio or Oxford Nanopore will be required to determine the structure of AbGRI1 in the isolates containing this island.

### The context of the *aphA1b* gene

**Figure 2:**
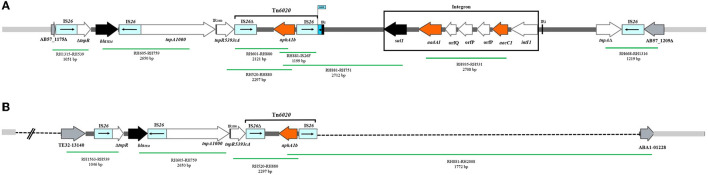
The AbGRI2 variants found in this study. **(A)** Schematic of the AbGRI2-1. **(B)** Schematic of the AbGRI2-12b. The central dark gray line represents the resistance island. The light gray line indicates the adjacent chromosomal sequence. The arrows indicate the extent and orientation of genes and open reading frames. The aminoglycoside resistance genes are orange and other resistance genes are black with the name below. The vertical bars indicate IRs. The dashed lines in AbGRI2-12b represent the extent of deletions relative to AbGRI2-1. The primers used to link sequences are indicated beneath each green line, along with the predicted amplicon size. Figure made adapted from Blackwell et al. (2017).

In all the 51 GC2 isolates containing *aphA1b* (52.5%), this gene was located in Tn*6020*, and the Tn*6020* in these isolates was carried by either AbGRI2-1 (one isolate, 1.03%) or AbGRI2-12b resistance islands (50 isolates, 51.5%). The structure of the AbGRI2-1 and AbGRI2-12b resistance islands is shown in Figure 2.

### The context of *aacC1* and *aadA1* genes

The *aacC1* and *aadA1* genes were found together only in one isolate containing AbGRI2-1 resistance island (Supplementary Table 4).

### The context of the *armA* gene

**Figure 3:**
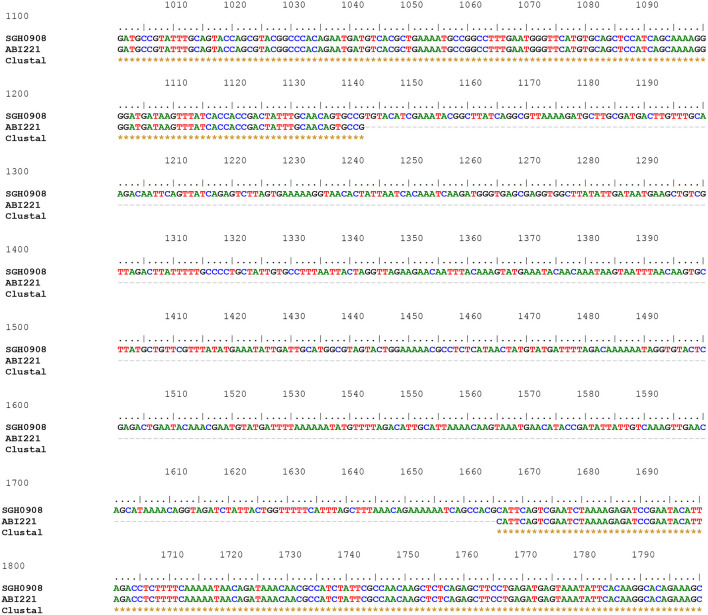
The sequence of Δ*atr*-ISAba24 segment missing 523 bp of the *repAciN* gene in the isolates containing AbGRI3_ABI221_ (accession number QQ341795) compared with the sequence of Δ*atr*-ISAba24 in SGH0908 containing AbGRI3-2i (accession number KX011026). Three rows are shown: the first row shows a part of the Δ*atr*-ISAba24 sequence in the SGH0908 isolate, and the second row shows the Δ*atr*-ISAba24 sequence missing 523 bp of the *repAciN* gene in AbGRI3_ABI221_. The dashed line in the middle row from nucleotide numbers 1142 to 1665 indicates the absence of 523 nucleotides in the isolates containing AbGRI3_ABI221_. Asterisks in the last row indicate the same nucleotides.

**Figure 4:**
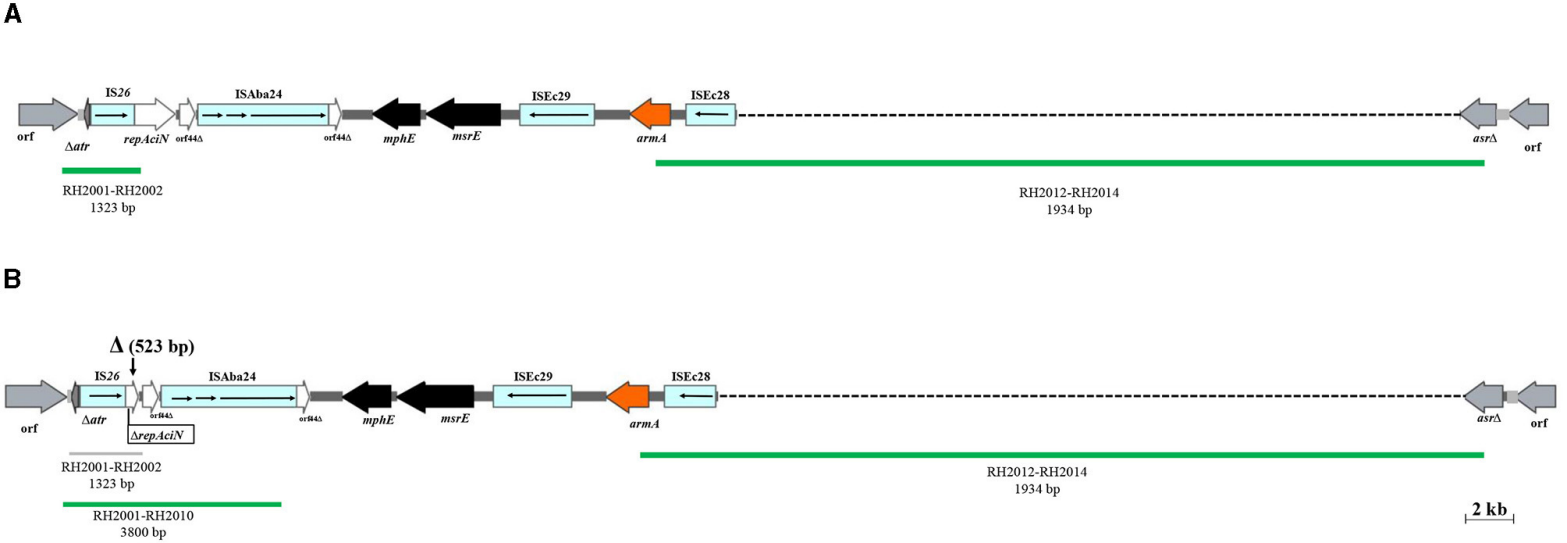
The AbGRI3 variants found in this study. **(A)** Schematic of the AbGRI3-4. (**B)** Schematic of the AbGRI3_ABI221_.The central dark gray line represents the resistance island. The light gray line indicates the adjacent chromosomal sequence. Arrows indicate the extent and orientation of genes and open reading frames. The aminoglycoside resistance genes are orange and other resistance genes are black with the name below. Vertical bars indicate IRs and the vertical arrows indicate the location of deletion (Δ) found in the AbGRI3_ABI221_ in this study. Dashed lines in AbGRI3-4 and AbGRI3_ABI221_ represent the extent of deletions relative to AbGRI3-2i and AbGRI3-4, respectively. The positive and negative result for each reaction, which was performed to detect AbGRI3 variants are shown by green and gray lines, respectively, with their respective primers and predicted amplicon sizes beneath each line. Figure made adapted from Blackwell et al. (2017).

Of the 88 *armA*-harboring isolates, 71 (80.7%) carried AbGRI3-4. In the remaining 17 isolates (19.3%), a new variant of AbGRI3 resistance islands was found, which was called AbGRI3_ABI221_ (Supplementary Table 4). The PCR for linking the left end of AbGRI3 to ISAba24 (Δ1atr-ISAba24) produced a larger amplicon than expected 3.8 kb (Blackwell, 2017). It was found that AbGRI3_ABI221_ is identical to AbGRI3-4 except for missing a 523 bp segment from the left-hand side of the island in our laboratory (ABI221) previously. The sequence of this amplicon was submitted to the NCBI GenBank database under the accession number QQ341795. The sequence of Δ*atr*-ISAba24 segment missing 523 bp of the *repAciN* gene (from nucleotide number 1,142 to 1,665) in the isolates containing AbGRI3_ABI221_ (accession number QQ341795) compared with the sequence of Δ*atr*-ISAba24 in SGH0908 containing AbGRI3-2i (accession number KX011026) is shown in Figure 3. The genetic structure of the AbGRI3-4 and AbGRI3_ABI221_ resistance islands is shown in Figure 4.

### The context of *aphA6* gene

Of the 55 isolates containing the *aphA6* gene (56.7%), the *aphA6* gene was flanked by ISAba125 in 30 isolates (54.5%); i.e., the *aphA6* gene is located in Tn*aphA6*. The amplicon for PCRs linking the *aphA6* to the backbone of RepAci6 plasmids on the right and left was amplified in 20 isolates of those containing Tn*aphA6* (66.6%), and also, PCRs for repeated sequences 1, 2, and 3 produced the expected amplicons, suggesting the presence of a RepAci6 plasmid (Supplementary Table 4).

### The context of the *aadB* gene

In all isolates containing the *aadB* (44.4%), this gene was carried by pRAY^*^. The class 1 integron was not detected in any isolate containing the *aadB* gene (Supplementary Table 4).

## Discussion

Aminoglycosides are used as therapeutic options when *A. baumannii* infections are resistant to carbapenems. However, the development of resistance to aminoglycosides has been described in many countries worldwide (Nemec et al., 2004; Nie et al., 2014; Kishk et al., 2021). Despite the characterization of MGEs carrying aminoglycoside resistance genes in GC2 *A. baumannii* in previous studies (Nigro et al., 2011, 2013, 2015; Hamidian et al., 2012; Nigro, 2014; Nigro and Hall, 2016; Blackwell, 2017; Blackwell et al., 2017), much less is known about their dissemination in the Middle East region, including Iran (Aris et al., 2019; Douraghi et al., 2020). This study examined the presence of MGEs associated with aminoglycoside resistance genes among GC2 isolates in Tehran, Iran. It is noteworthy that in the present study, the MGEs were detected in the isolates recovered from the patients admitted to different hospitals in different time periods. More than half of GC2 isolates were resistant to all tested aminoglycosides, and all contained at least one aminoglycoside resistance gene. Distinct aminoglycoside resistance profiles were found among the isolates. The *armA* gene was detected in all the isolates in group 1 (resistant to all aminoglycosides) and group 2 (resistant to all aminoglycosides except neomycin), respectively. It was revealed that all of the isolates containing the *armA* were located within the AbGRI3 resistance island. The chromosomally located islands carrying the *armA* gene, named AbGRI3 in 2017 (Blackwell et al., 2017), were described in the USA in 2014 (Wright et al., 2014). Consistent with the results of the current study, Blackwell detected the *armA* gene in 15 out of 20 GC2 *A. baumannii* isolates from Singapore, and they revealed that the *armA* gene is located in AbGRI3, in all the isolates containing this gene (Blackwell et al., 2017). Tn*6020* was first characterized in GC1 *A. baumannii* (Post and Hall, 2009) and then in GC2 isolates from Australia (Nigro et al., 2011). In this study, *aphA1b* was located within Tn*6020* in all the GC2 isolates containing this gene, and all Tn*6020* that were detected were carried by either AbGRI2-1 or AbGRI2-12b (Blackwell, 2017) resistance islands. While only 15% of GC2 isolates from Singapore contained AbGRI2-12b, this island was detected in nearly half of the isolates examined in the present study. The AbGRI2-1 resistance island harboring a class 1 integron (containing *aacC1* adjacent to *aadA1*) was only detected in one of the isolates tested, while it was present in 21.6 and 25% of GC2 isolates examined in Australia and Singapore, respectively (Nigro, 2014; Blackwell, 2017). The class 1 integron carrying the *aacC1* gene cassette is prevalent in GC1 isolates, but it is also found in GC2 isolates tested in Australia (Nigro et al., 2013). In GC1 strains, the class 1 integron carrying the *aacC1* gene cassette is usually located within an AbaR resistance island (Post et al., 2010, 2012; Krizova et al., 2011). However, the studies focusing on aminoglycoside resistance in GC2 isolates collected from Australia demonstrated that only a subgroup of GC2 isolates contained the *aacC1* gene cassette along with Tn*6020* (Post et al., 2010; Nigro et al., 2011). In this study, nearly half of the isolates contained AbGRI1 resistance island (carrying *strA* and *strB* resistance genes); however, all the GC2 isolates examined in Australia (Nigro and Hall, 2016), and 82.4% of the GC2 isolates investigated in South Korea carried AbGRI1 (Kim et al., 2017). Of all GC2 isolates which were resistant to amikacin, more than half contained the *aphA6* gene. Furthermore, out of the isolates carrying *aphA6*, more than half contained Tn*aphA6*; of them, 66.6% were harbored by RepAci6 plasmids. Tn*aphA6* was first identified in the Australian isolates belonging to GC2, and subsequently in a GC1 isolate from Australia (Nigro et al., 2011; Hamidian et al., 2014) and United States (Rao et al., 2018). The RepAci6 plasmids harboring Tn*aphA6* have been identified in *A. baumannii* isolates from Australia (Hamidian et al., 2014; Nigro and Hall, 2014). These plasmids were detected in GC1 *A. baumannii* isolates in Iran (Aris et al., 2019; Douraghi et al., 2021); however, there was no study to investigate their presence in GC2 isolates from Iran. In this study, it was revealed that more than half of GC2 isolates contain the RepAci6 plasmids carrying Tn*aphA6*. All the GC2 isolates carrying the *aadB* gene contained pRAY^*^. The *aadB* gene cassette was originally characterized in a class 1 integron (Cameron et al., 1986; Recchia and Hall, 1995); however, it was subsequently found at a secondary location on plasmid pRAY (Segal and Elisha, 1997, 1999). The class 1 integron carrying *aadB* was not found in GC2 isolates in the current study, which is consistent with the previous study by Nemec and Nigro (Nemec et al., 2004; Nigro et al., 2011). Hence, pRAY^*^ had a significant role in carrying the *aadB* gene in the isolates tested.

## Conclusion

This study provides the evidence for dissemination of aminoglycoside resistance genes through MGEs in GC2 *A. baumannii* isolated from Iran as the second-largest country in the Middle East. In addition, this finding suggests that the strains carrying these MGEs are not restricted to a particular geographical region, and they are globally distributed. The results obtained in this study will underpin future studies of GC2 strains from other countries.

## Data availability statement

The datasets presented in this study can be found in online repositories. The names of the repository/repositories and accession number(s) can be found in the article/Supplementary material.

## Ethics statement

The Ethics Committee of Tehran University of Medical Sciences approved the study IR.TUMS.SPH.REC.1397.291. All patients have signed the informed consent for giving the required specimens for research. Parents/legal guardians provided written informed consent for their children under the age of 18 years to participate in this study.

## Author contributions

GN performed the microbiological and molecular experiments and wrote and revised the manuscript. MT and MR were the advisors of the project. RG and SG did the molecular experiments. AS and AA were the clinical advisors of the project. AN was involved in the collection of isolates from the hospital. MD conceptualized, designed, coordinated, supported this study, contributed to the acquisition of fund, the analysis and interpretation of data, and performed the revision of the manuscript. All authors have read and agreed to the published version of the manuscript.
