# Supplementary material for: Mobile genetic elements carrying aminoglycoside resistance genes in Acinetobacter baumannii isolates belonging to global clone 2
Source: Front Microbiol. 2023 May 5;14:1172861. doi: 10.3389/fmicb.2023.1172861 (PMC10196456; doi:10.3389/fmicb.2023.1172861)
Supplement: Supplementary file 1 [file Table_1.pdf]

Supplementary Material

Table S1. The primers used for sequencing by primer walking strategy

**Primer**	**Sequence (5'-3')**	**Reference**
RH2001	GGAGTTGGTTTTGGTACAGCA	Blackwell, 2017
RH2010	TTTCGTGACACTCTCGCTTG	Blackwell, 2017
F1	AGTGCGGCCCCGGCTGTCGACG	This study
R1	GATAACATCAACGCGCGGCAGC	This study
F2	TATTCGCCAACAAGCTCTCAG	This study
R2	TAATGCAGTGGCTGATGGGCG	This study

Table S2. Primer pairs used to determine the context of aminoglycoside resistance genes.

PCR	Primer	Sequence (5'-3')	Annealing temperature (° C)	Amplicon length (bp)	Reference
comM	RH927<br>RH928	CAACCCTGTCTTTGCATTTG<br>GCCAGCAAGCTCAGCATAA	59	880	Nigro, 2014
comM–AbGRI1 (J1)	RH927<br>RH792	CAACCCTGTCTTTGCATTTG<br>TTCGAGCTTGAAAACTGCAC	60	846	Nigro, 2014
AbGRI1–*comM* (J2)	RH928<br>RH916	GCCAGCAAGCTCAGCATAA<br>CCCAAATACTGCCATGTTGA	60	796	Nigro, 2014
orf4b-*comM*	RH594<br>RH928	GGCGGATTATCAGTTGTTTCA<br>GCCAGCAAGCTCAGCATAA	60	1844	Nigro, 2014
tniBΔ-*tniEΔ*	RH910<br>RH587	GCGATAGTGAACGGATTGAGA<br>TTGCCCATTAAGCACAACAG	60	560	Nigro, 2014
tniE-*tniB*	RH910<br>RH587	GCGATAGTGAACGGATTGAGA<br>TTGCCCATTAAGCACAACAG	60	3410	Nigro, 2014
tniD-*tniB*	RH910<br>RH584	GCGATAGTGAACGGATTGAGA<br>TCAATATGCCTCGCTCCACT	60	2010	Nigro, 2014
uspA- *tniD*	RH583<br>RH919	TCCTGTCTCTCGTGTAGCAAT<br>TGTCAAAAATTATTGCATGT	60	3577	Nigro, 2014
Tn-*comM*	RH791<br>RH909	TGCTGCAATGAGCTGAAAGT<br>GCGATTCAAAATATCGGTCAA	60	3119	Nigro, 2014
uspA-sup	RH793<br>RH771	CCCAAGAGAGCTGATTTTGC<br>TGTAAAATCTGGTGGTCGTAC	60	3267	Nigro, 2014
ISAba1-*sul2*	ISAba1B<br>sul2-R	CATGTAAACCAATGCTCACC<br>ATGCCGGGATCAAGGACAAG	60	1125	Nigro, 2014
strA-*strB*	strA-F<br>strB-R	CTTGGTGATAACGGCAATTC<br>GGATCGTAGAACATATTGGC	58	1190	Nigro, 2014
strA-*comM*	strA-R<br>RH928	CCAATCGCAGATAGAAGGC<br>GCCAGCAAGCTCAGCATAA	60	3509	Nigro, 2014
strB-orf4b	strB-R<br>RH599	GGATCGTAGAACATATTGGC<br>ATACTGTTTCAAAAACTGATGAA	60	2620	Nigro, 2014
CR2	LECR2<br>RECR2	CACTGGCTGGCAATGTCTAG<br>CTTTGGACCGCAGTTGACTC	60	1793	Nigro, 2014
**CR2- *strB***	strB-F<br>RECR2	ATCGTCAAGGGATTGAAACC<br>CTTTGGACCGCAGTTGACTC	60	2962	Nigro, 2014
tetA(B)- tetR(B)	tetB-R<br>RH893	GTAATGGGCCAATAACACCG<br>AGAAGGCTGGCTCTGCACCT	60	1693	Nigro, 2014
***tetR(B)*-CR2**	RH892<br>LECR2	ACAGCGCATTAGAGCTGCTT<br>CACTGGCTGGCAATGTCTAG	60	2812	Nigro, 2014
orf6-orf7	RH1302<br>RH1303	CAAATCGGGAAGGTTCAAAA<br>CGGGAAAATTACTGCGATTG	60	1573	Nigro, 2014
int-orf11	RH1306<br>RH1307	GCATACTCATGTGGTTTAAGACT TG<br>TTAATTGCTTCATCATTTGAGC	60	1638	Nigro, 2014
orf9-*tniCb*	RH597<br>RH792	TTTGAAGAAATTGAGCATGAGG<br>TTCGAGCTTGAAAACTGCAC	60	1566	Nigro, 2014
AB57_1175 - t*npR*$_1$	RH1315<br>RH539	AGGAGATCTTCTTGGCAGTCA<br>CCAGCCCTTCCCGATCTGTTG	60	1051	Nigro, 2014
bla$_{TEM}$- *tnpA*$_{1000}$	RH605<br>RH759	TTTCGTGTCGCCCTTATTCC<br>GCCAGCTCATTTACCTTGCCGA	60	2650	Nigro, 2014
tnpR$_{5393}$*c*-*aphA1*	RH520<br>RH880	CATGGCCCAGCGCGATACTTCAG<br>CAACGGGAAACGTCTTGCTC	60	2297	Nigro, 2014
aphA1- *sul1*	RH881<br>RH751	ATTCGTGATTGCGCCTGAG<br>GCGGAACTTCACGCGATC	60	2712	Nigro, 2014
tnpA$_{21}$-AB57_1209	RH668<br>RH1316	CACCAGAACCGCCTGCTCAA<br>CATCTGCCATCCAGTTTGTG	60	1219	Nigro, 2014
TE32_13140-*tnpR1*	RH1563<br>RH539	ATAGATCGGCTTCGGACTCA<br>CCAGCCCTTCCCGATCTGTTG	60	1046	Blackwell, 2017
aphA1b-ABA1_01228	RH881<br>RH2008	ATTCGTGATTGCGCCTGAG<br>TGATGACTTCCATTAAAGCCTGT	60	1581[a]	Blackwell, 2017

PCR	Primer	Sequence (5'-3')	Annealing temperature (° C)	Amplicon length (bp)	Reference
IS*26*-*aphA1*	RH601	GATGGAGCTGCACATGAACC	60	2121	Nigro, 2014
	RH880	CAACGGGAAACGTCTTGCTC			
aphA1- IS*26*	RH881	ATTCGTGATTGCGCCTGAG	60	1199	Nigro, 2014
	IS26F	ACCTTTGATGGTGGCGTAAG			
atr	RH2001	GGAGTTGGTTTTGGTACAGCA	60	400	Blackwell, 2017
	RH2004	AATGTGGTTGGCGGTTTTTA			
Δatr-*repAciN*	RH2001	GGAGTTGGTTTTGGTACAGCA	60	1323	Blackwell, 2017
	RH2002	TATAAGCCACCTCGCTCACC			
Δatr-ISAba24	RH2001	GGAGTTGGTTTTGGTACAGCA	60	1605[b]	Blackwell, 2017
	RH2010	TTTCGTGACACTCTCGCTTG			
armA-*asrΔ*	RH2012	TCCATTCCCTTCTCCTTTCC	60	1934	Blackwell, 2017
	RH2014	CCAAATACCGCCCACTCAAC			
aphA1b- *Δasr*	RH831	TATACCCATATAAATCAGCATCC	60	1203	Blackwell, 2017
	RH2005	CACTGATCTGCTGGCTTTCA			
aadB in pRAY	RH561	GGGAAGAATCAATACCGCAA	62	998	Nigro, 2014
	RH562	AATTTCACCCCAAACAATCG			
intI1	HS463a	CTGGATTTCGATCACGGCACG	58	472	Nigro, 2014
	HS464	ACATGCGTGTAAATCATCCGTCG			
ISAba125-*aphA6*	RH573	AAGAAGGCTTTTCAGCCAGA	60	1427	Nigro, 2014
	aphA6R	GGACAATCAATAATAGCAAT			
aphA6-ISAba125	aphA6F	ATACAGAGACCACCATACAGT	60	1745	Nigro, 2014
	RH574	CAAACATGAGGTGCGACAGT			
Tn*aphA6*_L	RH1501	CTTGAGGAAGGGATGGTTGA	59	1930	Blackwell, 2017
	aphA6R	GGACAATCAATAATAGCAAT			
Tn*aphA6*_R	aphA6F	ATACAGAGACCACCATACAGT	59	2540	Blackwell, 2017
	RH1502	TTGCTTTAATCGGTGGTTCC			
repAci6	RH2045	AAGGTGACAGCAAGTACGTG	60	482	Blackwell, 2017
	RH2046	CGTGCGCCTCATTTCAACAT			
Repeated sequence 1	RH1398	TTTGACGTTGCTCTTGTTGC	60	987	Blackwell, 2017
	RH1399	TTCTCCCAAGTGGTCAGGTC			
Repeated sequence 2	RH1395	TCAAACGATGCAATGGAAGA	60	1373	Blackwell, 2017
	RH1394	TGGTTGGCAGAACAAGATGA			
Repeated sequence 3	RH1503	GAAGATCCAGAAGCGGGATA	60	1576	Blackwell, 2017
	RH1397	CCATGTTCTTTTCCACATGC			

a. Predicted size based on AbGRI2-12a. For AbGRI2-12b it is 1,772.

b. The size of this segment in the isolates containing AbGRI3$_{ABI221}$ is 3800 bp.

Table S3*. Results of disk diffusion for GC2 *A. baumannii* isolates recovered from Iranian hospital.

Isolate	Sm	Sp	Su	Tc	Km	Nm	CTX	CAZ	Gm	Cip	AK	Nx	Tm	Ne	Ipm	Mem	TIM	Rif	SAM	FEP	DOR	TZP	CRO	MIN	Dox	LVX	TS
ABS470	6	6	6	6	6	9	6	6	12	6	8	6	20	17	6	6	6	6	6	6	6	6	6	14	8	6	6
ABS495	6	6	6	6	6	10	6	6	12	6	10	6	17	17	6	6	6	6	10	6	6	8	6	15	6	6	6
ABS496	6	6	6	6	6	11	6	6	9	6	11	6	20	17	8	6	6	6	8	6	6	8	6	13	8	6	6
ABS534	6	6	13	6	6	18	6	6	6	6	6	6	6	6	6	6	6	8	10	6	6	7	6	7	6	7	6
ABS564	6	6	6	8	6	6	6	6	6	6	6	6	6	6	6	7	6	12	9	7	6	8	6	16	11	9	6
ABS565	6	6	6	6	6	6	6	6	6	6	9	6	6	13	6	6	6	12	14	8	6	8	6	16	10	10	6
ABS566	6	6	6	6	6	11	6	6	6	6	6	6	6	22	6	6	6	6	8	6	6	6	6	12	8	6	6
ABS567	11	6	10	6	6	22	6	6	6	6	6	6	6	6	6	6	6	12	10	6	6	8	6	15	10	8	6
ABS568	10	6	6	10	6	6	6	6	6	6	6	6	6	6	6	6	6	11	10	6	6	7	6	16	13	6	6
ABS569	6	6	6	6	6	6	6	6	6	6	6	6	6	6	6	6	6	11	8	10	6	6	6	12	10	9	6
ABS570	6	6	6	6	6	6	6	6	6	6	6	6	6	6	6	6	6	12	7	6	6	7	6	15	10	9	6
ABS571	11	6	6	6	6	6	6	6	6	6	6	6	6	6	6	6	6	12	8	6	6	7	6	14	10	6	6
ABS572	11	7	6	9	6	6	6	6	6	6	6	6	6	6	6	6	6	10	8	6	6	9	6	12	10	6	6
ABS573	6	6	6	6	6	6	6	6	6	6	6	6	6	6	10	6	6	13	14	7	6	12	6	7	6	7	6
ABS574	11	6	6	10	6	10	6	6	6	6	6	6	6	6	6	6	6	11	6	7	6	7	6	14	12	6	6
ABS575	11	6	6	10	6	10	6	6	6	6	6	6	6	6	6	6	6	12	6	7	6	7	6	16	14	10	6
ABS577	6	6	6	6	6	6	6	6	10	6	6	6	6	6	6	6	6	7	8	6	6	7	6	16	10	8	6
ABS580	6	6	23	7	6	6	6	6	10	6	15	6	6	11	6	6	6	8	7	6	6	7	6	15	11	8	12
ABS581	6	6	6	8	6	21	6	6	6	6	6	6	6	6	6	6	6	7	10	6	6	10	6	14	10	8	6
ABS582	11	7	21	8	6	6	6	6	11	6	6	6	6	6	6	6	6	6	6	6	6	6	6	15	13	8	12
ABS583	6	6	6	8	6	6	6	6	12	6	6	6	6	6	6	6	6	9	12	6	6	6	6	20	10	8	6
ABS588	6	6	6	6	6	6	6	6	6	6	14	6	6	6	6	6	6	10	10	8	6	10	6	17	12	9	6
ABS593	13	10	6	10	6	6	6	6	6	6	6	6	6	6	6	6	6	6	10	10	6	10	6	16	13	6	6
ABS594	6	6	6	6	6	20	6	6	6	6	6	6	6	6	6	6	6	11	11	6	6	10	6	12	6	8	6

Isolate	Sm	Sp	Su	Tc	Km	Nm	CTX	CAZ	Gm	Cip	AK	Nx	Tm	Ne	Ipm	Mem	TIM	Rif	SAM	FEP	DOR	TZP	CRO	MIN	Dox	LVX	TS
ABS614	6	6	6	6	6	6	6	6	12	6	6	6	6	6	6	6	6	10	10	9	6	10	6	17	11	10	6
ABM304	6	6	6	11	6	6	6	6	6	6	6	6	6	6	6	6	6	9	7	7	6	6	6	15	10	7	6
ABM305	12	7	6	9	6	8	6	6	6	6	6	6	6	6	6	6	6	10	8	7	6	7	6	15	7	6	6
ABM310	6	6	6	8	6	6	6	6	6	6	6	6	6	6	6	6	6	6	8	6	6	8	6	14	12	6	6
ABM313	11	8	20	11	6	6	6	6	6	6	6	6	6	6	6	6	6	11	9	7	6	10	6	15	14	6	10
ABM315	12	7	6	11	6	6	6	6	6	6	6	6	6	6	6	6	6	10	6	6	6	8	6	13	11	6	6
ABM316	15	15	23	10	6	20	6	6	6	6	6	6	6	6	6	6	6	11	12	7	6	10	6	18	15	6	12
ABM319	6	6	6	11	6	12	6	6	11	6	13	6	6	17	6	6	6	11	6	6	6	6	6	19	14	6	6
ABM322	13	6	20	10	6	20	6	6	6	6	6	6	6	6	6	6	6	12	8	8	6	9	6	18	15	6	17
ABM323	13	6	25	11	6	20	6	6	6	6	6	6	6	6	6	6	6	15	10	10	6	6	6	20	15	6	16
ABM324	13	8	6	6	6	17	6	6	6	6	6	6	6	6	6	6	6	10	8	8	6	6	6	16	11	6	6
ABM329	6	6	6	6	6	21	6	6	6	6	6	6	6	6	6	6	6	10	8	8	6	6	6	10	6	8	6
ABM331	13	6	6	6	6	6	6	6	6	6	6	6	6	6	6	6	6	10	8	8	6	6	6	15	11	6	6
ABM334	6	6	6	10	6	6	6	6	11	6	6	6	6	6	6	6	6	10	8	8	6	6	6	18	17	10	6
ABM336	6	6	6	6	6	8	6	6	10	6	6	6	16	17	6	6	6	6	8	6	6	6	6	10	6	6	6
ABM337	6	6	6	12	6	8	6	6	10	6	15	6	6	6	6	6	6	10	6	7	7	7	6	19	16	10	6
ABM338	6	6	6	6	6	17	6	6	6	6	6	6	6	6	6	6	6	10	8	6	8	8	6	12	6	10	6
ABM341	12	9	6	10	6	6	6	6	12	6	6	6	6	6	6	6	6	10	8	6	6	6	6	16	11	6	6
ABM342	9	6	6	10	6	17	6	6	12	6	6	6	6	10	6	6	6	9	8	8	6	6	6	15	12	8	6
ABM343	15	15	14	10	6	17	6	6	12	6	6	6	6	12	6	6	6	10	8	8	6	6	6	16	12	6	6
ABM345	6	6	6	6	6	17	6	6	12	6	6	6	6	6	6	6	6	10	8	6	6	6	6	10	6	6	6
ABM346	15	15	27	6	6	17	6	6	6	6	6	6	6	6	6	6	6	10	8	8	6	6	6	16	12	6	16
ABM366	13	12	6	8	6	6	6	6	6	6	6	6	6	6	6	6	6	9	8	7	6	6	6	16	12	6	6
ABM368	13	12	6	8	6	6	6	6	6	6	14	6	6	6	6	6	6	9	11	8	6	6	6	16	15	11	6
ABM377	6	6	6	7	6	8	6	6	12	6	6	6	6	6	6	6	6	10	8	6	6	6	6	16	12	7	6
ABM378	9	6	6	13	6	6	6	6	6	6	6	6	6	17	6	6	6	10	9	6	6	9	6	20	17	6	6
ABM379	14	14	6	6	6	6	6	6	6	6	6	6	6	6	6	6	6	9	8	7	6	6	6	12	8	6	6
ABM380	15	15	6	6	6	6	6	6	6	6	6	6	6	6	6	6	6	9	8	7	6	6	6	16	11	6	6
ABM382	7	6	6	8	6	6	6	6	6	6	6	6	6	6	6	6	6	9	7	6	6	6	6	16	10	8	6
ABM390	6	6	22	6	6	20	6	6	10	6	6	6	12	6	6	6	6	10	11	6	6	6	6	10	6	6	13

Isolate	Sm	Sp	Su	Tc	Km	Nm	CTX	CAZ	Gm	Cip	AK	Nx	Tm	Ne	Ipm	Mem	TIM	Rif	SAM	FEP	DOR	TZP	CRO	MIN	Dox	LVX	TS
ABM391	6	6	6	10	6	6	6	6	6	6	6	6	6	6	6	6	6	9	8	7	7	6	6	15	10	7	6
ABM392	6	6	12	6	6	6	6	6	10	6	14	6	6	16	6	6	6	10	7	6	6	6	6	19	17	6	6
ABM393	7	6	6	10	6	8	6	6	12	6	6	6	12	6	6	6	6	10	8	7	6	6	6	17	12	7	6
ABM395	11	6	6	10	6	8	6	6	12	6	6	6	6	6	6	6	6	10	8	7	6	6	6	14	10	6	6
ABM399	10	6	6	10	6	8	6	6	12	6	6	6	6	6	6	6	6	10	8	7	6	6	6	15	10	7	6
ABM402	11	6	6	8	6	6	6	6	6	6	6	6	6	6	6	6	6	10	9	8	6	6	6	17	13	6	6
ABM428	11	6	6	6	6	17	6	6	12	6	6	6	6	6	6	6	6	10	9	8	6	6	6	16	13	6	6
ABM429	6	6	6	6	6	6	6	6	6	6	15	6	6	6	6	6	6	10	6	6	6	6	6	17	10	11	6
ABM430	6	6	6	6	6	20	6	6	11	6	6	6	6	6	6	6	6	10	9	8	6	6	6	10	6	8	6
ABM432	11	6	6	7	6	8	6	6	6	6	6	6	6	6	6	6	6	10	9	8	6	6	6	16	13	6	6
ABM433	6	6	22	6	6	20	6	6	12	6	6	6	6	6	6	6	6	10	10	8	6	6	6	12	6	8	13
ABM434	6	7	22	6	6	20	6	6	12	6	6	6	6	6	6	6	6	10	12	9	6	6	6	10	6	6	11
ABM435	11	6	6	10	6	6	6	6	6	6	6	6	6	6	6	6	6	10	9	8	6	9	6	17	14	6	6
ABM438	11	6	26	13	6	6	6	6	12	6	6	6	6	6	6	6	6	10	9	10	6	8	6	21	20	11	16
ABM440	6	6	6	6	6	6	6	6	6	6	6	6	12	6	6	6	6	10	10	8	6	9	6	12	6	7	6
ABM441	6	7	25	6	6	20	6	6	12	6	6	6	6	6	6	6	6	10	13	8	6	6	6	10	6	8	12
ABM442	6	6	6	6	6	20	6	6	12	6	15	6	6	13	6	6	6	6	10	8	6	6	6	12	6	9	6
ABM444	6	6	6	6	6	21	6	6	12	6	12	6	6	6	6	6	6	6	10	6	6	9	6	10	6	9	6
ABM445	6	6	6	6	6	6	6	6	6	6	13	6	6	6	6	6	6	10	11	9	6	9	6	10	6	8	6
ABM459	6	6	6	6	6	20	6	6	6	6	6	6	6	6	6	6	6	6	11	6	6	10	6	10	6	6	6
ABM460	6	6	23	6	6	20	6	6	6	6	14	6	6	6	6	6	6	10	11	6	6	10	6	10	6	8	10
ABM461	6	6	23	6	6	20	6	6	12	6	6	6	6	6	6	6	6	10	11	6	6	9	6	10	6	6	10
ABM462	6	6	23	6	6	20	6	6	6	6	6	6	6	6	6	6	6	10	13	6	6	10	6	10	6	8	6
ABM463	11	6	6	6	6	21	6	6	6	6	6	6	6	6	6	6	6	10	11	6	6	10	6	17	13	6	6
ABM465	11	6	6	10	6	6	6	6	6	6	12	6	6	6	6	6	6	10	10	6	6	10	6	17	15	8	6
ABM466	6	6	21	6	6	20	6	6	6	6	13	6	6	6	6	6	6	10	11	6	6	10	6	11	6	7	10
ABM467	6	6	6	6	6	20	6	6	12	6	14	6	6	6	6	6	6	10	12	6	6	9	6	10	6	8	6
ABM468	6	6	14	6	6	20	6	6	6	6	6	6	6	6	6	6	6	10	11	6	6	10	6	10	6	6	6
ABM469	6	6	21	6	6	20	6	6	6	6	6	6	6	6	8	6	6	10	12	6	6	6	6	10	6	6	6

Isolate	Sm	Sp	Su	Tc	Km	Nm	CTX	CAZ	Gm	Cip	AK	Nx	Tm	Ne	Ipm	Mem	TIM	Rif	SAM	FEP	DOR	TZP	CRO	MIN	Dox	LVX	TS
ABM471	6	6	23	6	6	20	6	6	6	6	6	6	6	6	6	6	6	10	12	6	6	10	6	11	6	6	10
ABM472	6	6	6	12	6	13	6	6	11	6	13	6	20	17	6	6	6	12	10	6	6	9	6	12	20	6	6
ABM473	6	6	6	6	6	20	6	6	6	6	6	6	6	6	6	6	6	6	10	6	6	10	6	10	6	6	6
ABM474	6	6	23	6	6	20	6	6	6	6	6	6	6	6	8	6	6	10	13	6	6	10	6	10	6	6	10
ABM475	6	6	23	6	6	20	6	6	6	6	6	6	6	6	6	6	6	10	12	6	6	10	6	10	6	7	10
ABM476	6	6	6	10	6	9	6	6	6	6	6	6	6	6	6	6	6	10	6	6	6	6	6	16	13	6	6
ABH001	6	6	6	6	6	6	6	6	6	6	6	6	6	6	6	6	6	10	8	6	6	6	6	13	6	6	6
ABH003	6	6	6	6	6	22	6	6	6	6	6	6	6	6	6	6	6	11	15	9	6	6	6	13	6	12	6
ABH006	6	6	6	6	6	6	6	6	6	6	6	6	6	6	6	6	6	10	12	6	6	6	6	6	6	6	6
ABH007	6	6	6	6	6	21	6	6	6	6	6	6	6	6	6	6	6	6	10	6	6	6	6	6	6	6	6
ABH013	6	6	6	6	6	6	6	6	6	6	6	6	6	6	6	6	6	10	10	6	6	6	6	13	6	6	6
ABH014	10	6	30	6	6	21	6	6	6	6	6	6	6	6	6	6	6	13	9	6	6	6	6	13	6	6	6
ABH019	6	6	6	6	6	6	6	6	6	6	6	6	6	6	6	6	6	6	10	6	6	6	6	13	6	6	6
ABH065	6	6	6	12	6	6	6	6	6	6	6	6	6	6	6	6	6	6	6	6	6	6	6	13	6	6	6

* Sm: Streptomycin, Sp: Spectinomycin, Su: Sulfamethoxazole, Tc: Tetracycline, Km: Kanamycin, Nm: Neomycin, CTX: Cefotaxime, CAZ: Ceftazidime, Gm: Gentamicin, Cip: Ciprofloxacin, AK: Amikacin, Nx: Nalidixic Acid, Tm: Tobramycin, Ne: Netilmicin, Ipm: Imipenem, Mem: Meropenem, TIM: Timentin (Ticarcillin-clavulanic acid), Rif: Rifampicin, SAM: Ampicillin-sulbactam, FEP: Cefepime, DOR: Doripenem, TZP: Piperacillin-Tazobactam, CRO: Ceftriaxone, MIN: Minocycline, DOX: Doxycycline, LVX: Levofloxacin, TS: Trimethoprim-sulfamethoxazole, Inhibition zone diameters highlighted white, light gray, and dark gray indicate susceptibility, intermediate susceptibility, and resistance, respectively.

Table S4. Characteristics of the GC2 isolates containing AbGRI2 and AbGRI3 resistance island.

**Isolate**	Hospital	Year	Ward	*aacA4*	*aac(6')-Im*	*aacC2*	*aacC1*	*aadA1*	*aphA1b*	IS26-*aphA1b*	*aphA1b*-IS26	AB57_1175 - $tnpR_1$	bla_{TEM}- $tnpA_{1000}$	$tnpR_{5393}c$- *aphA1b*	*aphA1*- *sul1*	$tnpA_{21}$-AB57_1209	TE32_13140-*tnpR1*	*aphA1b*-ABA1_01228	AbGRI2	*armA*	*atr*	*Δatr-repAciN*	*Δatr*-ISAba24	*armA-asrΔ*	*aphA1b-Δasr*	AbGRI3	*aadB*	pRAY	*intI1*	*aphA6*	ISAba125-*aph6*	*aph6*- ISAba125	Tn*aphA6*_L	Tn*aphA6*_R	*repAci6*	Repeated sequence 1	Repeated sequence 2	Repeated sequence 3
**ABS470**	H4	2018	NR	-	-	-	+	-	+	+	+	-	+	+	-	-	+	+	AbGRI2-12b	-	+	ND[5]	ND[6]	ND[7]	ND[8]	No AbGRI3	-	ND[9]	+	+	+	+	+	-	+	+	+	+
**ABS495**	H4	2018	General Surgery	-	-	-	+	-	+	+	+	-	+	+	-	-	+	+	AbGRI2-12b	-	+	ND	ND	ND	ND	No AbGRI3	-	ND	+	+	+	+	+	+	+	+	+	+
**ABS496**	H4	2018	General ICU	-	-	-	+	-	+	+	+	-	+	+	-	-	+	+	AbGRI2-12b	-	+	ND	ND	ND	ND	No AbGRI3	-	ND	+	+	+	+	+	+	+	+	+	+
**ABS534**	H4	2019	Neurology ICU	-	-	-	-	-	-	ND[1]	ND[2]	-	-	-	-	-	ND[3]	ND[4]	No AbGRI2	+	-	-	+	+	-	$AbGRI3_{ABI221}$	+	+	-	-	ND[10]	ND[11]	ND[12]	ND[13]	ND[14]	ND[15]	ND[16]	ND[17]
**ABS564**	H4	2019	General ICU	-	-	-	-	-	+	+	+	-	+	+	-	-	+	+	AbGRI2-12b	+	-	+	-	+	-	AbGRI3-4	-	ND	-	+	-	-	ND	ND	ND	ND	ND	ND
**ABS565**	H4	2019	Internal General	-	-	-	-	+	+	+	+	-	+	+	-	-	+	+	AbGRI2-12b	+	-	+	-	+	-	AbGRI3-4	+	+	-	+	+	+	+	+	+	+	+	+
**ABS566**	H4	2019	Heart ICU	-	-	-	+	-	+	+	+	-	+	+	-	-	+	+	AbGRI2-12b	-	+	ND	ND	ND	ND	No AbGRI3	+	+	-	+	+	+	+	+	+	+	+	+
**ABS567**	H4	2019	General ICU	-	-	-	-	-	-	ND	ND	-	-	-	-	-	ND	ND	No AbGRI2	+	-	+	-	+	-	AbGRI3-4	-	ND	-	-	ND	ND	ND	ND	ND	ND	ND	ND
**ABS568**	H4	2019	Internal ICU	-	-	-	+	-	-	ND	ND	-	-	-	-	-	-	-	No AbGRI2	+	-	+	-	+	-	AbGRI3-4	+	+	-	+	+	+	+	+	+	+	+	+
**ABS569**	H4	2019	General ICU	-	-	-	-	-	+	+	+	-	+	+	-	-	+	+	AbGRI2-12b	+	-	+	-	+	-	AbGRI3-4	+	+	-	+	+	+	+	-	+	+	+	+
**ABS570**	H4	2019	General ICU	-	-	-	-	-	+	+	+	-	+	+	-	-	+	+	AbGRI2-12b	+	-	+	-	+	-	AbGRI3-4	+	+	-	+	+	+	+	-	+	+	+	+
**ABS571**	H4	2019	Heart CCU	-	-	-	-	-	+	+	+	-	+	+	-	-	+	+	AbGRI2-12b	+	-	+	-	+	-	AbGRI3-4	+	+	-	+	-	+	-	-	ND	ND	ND	ND
**ABS572**	H4	2019	Heart ICU	-	-	-	-	-	+	+	+	-	+	+	-	-	+	+	AbGRI2-12b	+	-	+	-	+	-	AbGRI3-4	+	+	-	+	-	-	ND	ND	ND	ND	ND	ND
**ABS573**	H4	2019	Internal ICU	-	-	-	-	-	+	+	+	-	+	+	-	-	+	+	AbGRI2-12b	+	-	+	-	+	-	AbGRI3-4	+	+	-	-	ND	ND	ND	ND	ND	ND	ND	ND
**ABS574**	H4	2019	Hematology oncology 3	-	-	-	-	-	+	+	+	-	+	+	-	-	+	+	AbGRI2-12b	+	-	+	-	+	-	AbGRI3-4	-	ND	-	-	ND	ND	ND	ND	ND	ND	ND	ND
**ABS575**	H4	2019	General ICU	-	-	-	-	-	+	+	+	-	+	+	-	-	+	+	AbGRI2-12b	+	-	+	-	+	-	AbGRI3-4	+	+	-	+	-	-	ND	ND	ND	ND	ND	ND
**ABS577**	H4	2019	General ICU	-	-	-	-	-	+	+	+	-	+	+	-	-	+	+	AbGRI2-12b	+	-	+	-	+	-	AbGRI3-4	+	+	-	+	+	+	+	+	+	+	+	+
**ABS580**	H4	2019	Surgery	-	-	-	-	-	+	+	+	-	+	+	-	-	+	+	AbGRI2-12b	+	-	+	-	+	-	AbGRI3-4	-	ND	-	+	+	+	-	-	ND	ND	ND	ND
**ABS581**	H4	2019	Internal ICU	-	-	-	-	-	-	ND	ND	-	-	-	-	-	ND	ND	No AbGRI2	+	-	+	-	+	-	AbGRI3-4	-	-	-	-	ND	ND	ND	ND	ND	ND	ND	ND
**ABS582**	H4	2019	Surgery	-	-	-	-	-	+	+	+	-	+	+	-	-	+	+	AbGRI2-12b	+	-	+	-	+	-	AbGRI3-4	+	+	-	+	+	+	+	+	+	+	+	+
**ABS583**	H4	2019	General ICU	-	-	-	-	-	+	+	+	-	+	+	-	-	+	+	AbGRI2-12b	+	-	+	-	+	-	AbGRI3-4	-	ND	-	+	+	+	-	+	+	+	+	+

**Isolate**	Hospital	Year	Ward	*aacA4*	*aac(6')-Im*	*aacC2*	*aacC1*	*aadA1*	*aphA1b*	IS*26*-*aphA1b*	*aphA1b*-IS*26*	AB57_1175 - *tnpR$_1$*	*bla$_{TEM}$*, *tnpA$_{1000}$*	*tnpR$_{5393c}$*- *aphA1b*	*aphA1*- *sul1*	*tnpA$_{21}$*-AB57_1209	TE32_13140-*tnpR1*	*aphA1b*-ABA1_01228	AbGRI2	*armA*	*atr*	Δ*atr*-*repAciN*	Δ*atr*-ISAba24	*armA*-Δ*asr*	*aphA1b*-Δ*asr*	AbGRI3	*aadB*	pRAY	*intI1*	*aphA6*	ISAba125-*aph6*	*aph6*- ISAba125	Tn*aphA6*_L	Tn*aphA6*_R	*repAci6*	Repeated sequence 1	Repeated sequence 2	Repeated sequence 3
**ABS588**	H5	2019	Burn 2	-	-	-	-	-	+	+	+	-	+	+	-	-	+	+	AbGRI2-12b	+	-	+	-	+	-	AbGRI3-4	+	+	-	+	-	+	+	-	+	+	+	+
**ABS593**	H5	2019	Burn 2	-	-	-	-	-	+	+	+	-	+	+	-	-	+	+	AbGRI2-12b	+	-	+	-	+	-	AbGRI3-4	+	+	-	+	+	+	+	+	+	+	+	+
**ABS594**	H5	2019	Burn 2	-	-	-	-	+	-	ND	ND	-	-	-	-	-	ND	ND	No AbGRI2	+	-	+	-	+	-	AbGRI3-4	+	+	-	+	+	+	+	+	+	+	+	+
**ABS614**	H5	2019	Burn 2	-	-	-	-	-	+	+	+	-	+	+	-	-	+	+	AbGRI2-12b	+	-	+	-	+	-	AbGRI3-4	+	+	-	+	+	+	-	+	+	+	+	+
**ABM304**	H1	2018	General ICU	-	-	-	-	-	+	+	+	-	+	+	-	-	+	+	AbGRI2-12b	+	-	+	-	+	-	AbGRI3-4	-	ND	-	-	ND	ND	ND	ND	ND	ND	ND	ND
**ABM305**	H1	2018	General ICU	-	-	-	-	-	+	+	+	-	+	+	-	-	+	+	AbGRI2-12b	+	-	+	-	+	-	AbGRI3-4	+	+	-	+	-	-	ND	ND	ND	ND	ND	ND
**ABM310**	H1	2018	Internal ICU	-	-	-	-	-	+	+	+	-	+	+	-	-	+	+	AbGRI2-12b	+	-	+	-	+	-	AbGRI3-4	-	ND	-	+	+	+	+	-	+	+	+	+
**ABM313**	H1	2019	General ICU	-	-	-	-	-	+	+	+	-	+	+	-	-	+	+	AbGRI2-12b	+	-	+	-	+	-	AbGRI3-4	-	ND	-	-	ND	ND	ND	ND	ND	ND	ND	ND
**ABM315**	H1	2019	NR	-	-	-	-	-	+	+	+	-	+	+	-	-	+	+	AbGRI2-12b	+	-	+	-	+	-	AbGRI3-4	+	+	-	-	ND	ND	ND	ND	ND	ND	ND	ND
**ABM316**	H1	2019	Internal ICU	-	-	-	-	-	-	ND	ND	-	-	-	-	-	ND	ND	No AbGRI2	+	-	+	-	+	-	AbGRI3-4	+	+	-	-	ND	ND	ND	ND	ND	ND	ND	ND
**ABM319**	H1	2019	Orthopedics	-	-	-	+	-	+	+	+	-	+	+	-	-	+	+	AbGRI2-12b	-	+	ND	ND	ND	ND	No AbGRI3	+	+	+	+	-	-	ND	ND	ND	ND	ND	ND
**ABM322**	H1	2019	ICU	-	-	-	-	-	-	ND	ND	-	-	-	-	-	ND	ND	No AbGRI2	+	-	+	-	+	-	AbGRI3-4	+	+	-	+	-	-	ND	ND	ND	ND	ND	ND
**ABM323**	H1	2019	CCU	-	-	-	-	-	-	ND	ND	-	-	-	-	-	ND	ND	No AbGRI2	+	-	+	-	+	-	AbGRI3-4	+	+	-	-	ND	ND	ND	ND	ND	ND	ND	ND
**ABM324**	H1	2019	Internal Ward	-	-	-	-	-	-	ND	ND	-	-	-	-	-	ND	ND	No AbGRI2	+	-	+	-	+	-	AbGRI3-4	+	+	-	-	ND	ND	ND	ND	ND	ND	ND	ND
**ABM329**	H1	2019	Outpatient	-	-	-	-	-	-	ND	ND	-	-	-	-	-	ND	ND	No AbGRI2	+	-	+	-	+	-	AbGRI3-4	+	+	-	-	ND	ND	ND	ND	ND	ND	ND	ND
**ABM331**	H1	2019	ICU	-	-	-	-	-	+	+	+	-	+	+	-	-	+	+	AbGRI2-12b	+	-	+	-	+	-	AbGRI3-4	-	ND	-	-	ND	ND	ND	ND	ND	ND	ND	ND
**ABM334**	H1	2019	ICU	-	-	-	-	-	+	+	+	-	+	+	-	-	+	+	AbGRI2-12b	+	-	+	-	+	-	AbGRI3-4	-	ND	-	-	ND	ND	ND	ND	ND	ND	ND	ND
**ABM336**	H1	2019	ICU	-	-	-	+	-	+	+	+	-	+	+	-	-	+	+	AbGRI2-12b	-	+	ND	ND	ND	ND	No AbGRI3	-	ND	-	+	+	+	+	-	+	+	+	+
**ABM337**	H1	2019	ICU	-	-	-	-	-	+	+	+	-	+	+	-	-	+	+	AbGRI2-12b	+	-	+	-	+	-	AbGRI3-4	+	+	-	+	-	-	ND	ND	ND	ND	ND	ND
**ABM338**	H1	2019	ICU	-	-	-	-	-	-	ND	ND	-	-	-	-	-	ND	ND	No AbGRI2	+	-	+	-	+	-	AbGRI3-4	-	ND	-	-	ND	ND	ND	ND	ND	ND	ND	ND
**ABM341**	H1	2019	Internal Ward	-	-	-	-	-	+	+	+	-	+	+	-	-	+	+	AbGRI2-12b	+	-	+	-	+	-	AbGRI3-4	+	+	-	+	-	+	+	+	+	+	+	+
**ABM342**	H1	2019	Surgery	-	-	-	-	-	-	ND	ND	-	-	-	-	-	ND	ND	No AbGRI2	+	-	+	-	+	-	AbGRI3-4	-	ND	-	-	ND	ND	ND	ND	ND	ND	ND	ND
**ABM343**	H1	2019	ICU	-	-	-	-	-	-	ND	ND	-	-	-	-	-	ND	ND	No AbGRI2	+	-	+	-	+	-	AbGRI3-4	-	ND	-	-	ND	ND	ND	ND	ND	ND	ND	ND
**ABM345**	H1	2019	EmergencyB	-	-	-	-	-	-	ND	ND	-	-	-	-	-	ND	ND	No AbGRI2	+	-	+	-	+	-	AbGRI3-4	+	+	-	-	ND	ND	ND	ND	ND	ND	ND	ND
**ABM346**	H1	2019	Internal Ward	-	-	-	-	-	-	ND	ND	-	-	-	-	-	ND	ND	No AbGRI2	+	-	+	-	+	-	AbGRI3-4	+	+	-	-	ND	ND	ND	ND	ND	ND	ND	ND
**ABM366**	H1	2019	Internal ICU	-	-	-	-	-	+	+	+	-	+	+	-	-	+	+	AbGRI2-12b	+	-	+	-	+	-	AbGRI3-4	+	+	-	+	-	+	+	+	+	+	+	+
**ABM368**	H1	2019	Internal ICU	-	-	-	-	-	+	+	+	-	+	+	-	-	+	+	AbGRI2-12b	+	-	+	-	+	-	AbGRI3-4	+	+	-	-	ND	ND	ND	ND	ND	ND	ND	ND
**ABM377**	H1	2019	Internal Ward	-	-	-	-	-	+	+	+	-	+	+	-	-	+	+	AbGRI2-12b	+	-	+	-	+	-	AbGRI3-4	+	+	-	+	+	+	+	+	+	+	+	+

**Isolate**	Hospital	Year	Ward	*aacA4*	*aac(6')-Im*	*aacC2*	*aacC1*	*aadA1*	*aphA1b*	IS26-*aphA1b*	*aphA1b*-IS26	AB57_1175 - *tnpR*$_1$	*bla*$_{TEM}$- *tnpA*$_{1000}$	*tnpR*$_{5393c}$- *aphA1b*	*aphA1*- *sul1*	*tnpA*$_{21}$-AB57_1209	TE32_13140-*tnpR1*	*aphA1b*-ABA1_01228	AbGRI2	*armA*	*atr*	Δ*atr*-*repAciN*	Δ*atr*-ISAba24	*armA*-*asr*Δ	*aphA1b*-Δ*asr*	AbGRI3	*aadB*	pRAY	*intI1*	*aphA6*	ISAba125-*aph6*	*aph6*- ISAba125	Tn*aphA6*_L	Tn*aphA6*_R	*repAci6*	Repeated sequence 1	Repeated sequence 2	Repeated sequence 3
**ABM378**	H1	2019	Surgery	-	-	-	+	+	+	+	+	+	+	+	+	+	ND	ND	AbGRI2-1	-	+	ND	ND	ND	ND	No AbGRI3	+	+	+	+	+	+	-	-	ND	ND	ND	ND
**ABM379**	H1	2019	ICU	-	-	-	-	-	+	+	+	-	+	+	-	-	+	+	AbGRI2-12b	+	-	+	-	+	-	AbGRI3-4	-	-	-	-	ND	ND	ND	ND	ND	ND	ND	ND
**ABM380**	H1	2019	ICU	-	-	-	-	-	+	+	+	-	+	+	-	-	+	+	AbGRI2-12b	+	-	+	-	+	-	AbGRI3-4	-	-	-	+	+	+	+	+	+	+	+	+
**ABM382**	H1	2019	ICU	-	-	-	-	-	+	+	+	-	+	+	-	-	+	+	AbGRI2-12b	+	-	+	-	+	-	AbGRI3-4	-	-	-	-	ND	ND	ND	ND	ND	ND	ND	ND
**ABM390**	H1	2019	Surgery	-	-	-	-	-	-	ND	ND	-	-	-	-	-	ND	ND	No AbGRI2	+	-	-	-	+	-	AbGRI3$_{ABI221}$	+	+	-	-	ND	ND	ND	ND	ND	ND	ND	ND
**ABM391**	H1	2019	ICU	-	-	-	-	-	+	+	+	-	+	+	-	-	+	+	AbGRI2-12b	+	-	+	-	+	-	AbGRI3-4	-	ND	-	-	ND	ND	ND	ND	ND	ND	ND	ND
**ABM392**	H1	2019	Emergency	-	-	-	+	-	+	+	+	-	+	+	-	-	+	+	AbGRI2-12b	-	+	ND	ND	ND	ND	No AbGRI3	+	+	+	+	-	-	ND	ND	ND	ND	ND	ND
**ABM393**	H1	2019	Internal Ward	-	-	-	-	-	+	+	+	-	+	+	-	-	+	+	AbGRI2-12b	+	-	+	-	+	-	AbGRI3-4	+	+	-	-	ND	ND	ND	ND	ND	ND	ND	ND
**ABM395**	H1	2019	ICU	-	-	-	-	-	+	+	+	-	+	+	-	-	+	+	AbGRI2-12b	+	-	+	-	+	-	AbGRI3-4	-	ND	-	-	ND	ND	ND	ND	ND	ND	ND	ND
**ABM399**	H1	2019	ICU	-	-	-	-	-	+	+	+	-	+	+	-	-	+	+	AbGRI2-12b	+	-	+	-	+	-	AbGRI3-4	+	+	-	-	ND	ND	ND	ND	ND	ND	ND	ND
**ABM402**	H1	2019	ICU	-	-	-	-	-	+	+	+	-	+	+	-	-	+	+	AbGRI2-12b	+	-	+	-	+	-	AbGRI3-4	-	ND	-	+	+	+	+	+	+	+	+	+
**ABM428**	H1	2019	Internal ICU	-	-	-	-	-	-	ND	ND	-	-	-	-	-	ND	ND	No AbGRI2	+	-	+	-	+	-	AbGRI3-4	-	ND	-	-	ND	ND	ND	ND	ND	ND	ND	ND
**ABM429**	H1	2019	ICU	-	-	-	-	-	+	+	+	-	+	+	-	-	+	+	AbGRI2-12b	+	-	+	-	+	-	AbGRI3-4	-	ND	-	-	ND	ND	ND	ND	ND	ND	ND	ND
**ABM430**	H1	2019	ICU	-	-	-	-	-	-	ND	ND	-	-	-	-	-	ND	ND	No AbGRI2	+	-	+	-	+	-	AbGRI3-4	-	ND	-	-	ND	ND	ND	ND	ND	ND	ND	ND
**ABM432**	H1	2019	Internal ICU	-	-	-	-	-	+	+	+	-	+	+	-	-	+	+	AbGRI2-12b	+	-	+	-	+	-	AbGRI3-4	+	+	-	-	ND	ND	ND	ND	ND	ND	ND	ND
**ABM433**	H1	2019	Internal Ward	-	-	-	-	-	-	ND	ND	-	-	-	-	-	ND	ND	No AbGRI2	+	-	+	-	+	-	AbGRI3-4	-	ND	-	+	-	-	ND	ND	ND	ND	ND	ND
**ABM434**	H1	2019	ICU	-	-	-	-	-	-	ND	ND	-	-	-	-	-	ND	ND	No AbGRI2	+	-	-	+	+	-	AbGRI3$_{ABI221}$	-	ND	-	+	-	-	ND	ND	ND	ND	ND	ND
**ABM435**	H1	2019	ICU	-	-	-	-	-	+	+	+	-	+	+	-	-	+	+	AbGRI2-12b	+	-	+	-	+	-	AbGRI3-4	+	+	-	-	ND	ND	ND	ND	ND	ND	ND	ND
**ABM438**	H1	2019	Surgery	-	-	-	-	-	+	+	+	-	+	+	-	-	+	+	AbGRI2-12b	+	-	+	-	+	-	AbGRI3-4	-	ND	-	+	+	+	+	+	+	+	+	+
**ABM440**	H1	2019	ICU	-	-	-	-	-	-	ND	ND	-	-	-	-	-	ND	ND	No AbGRI2	+	-	+	-	+	-	AbGRI3-4	-	ND	-	+	+	+	+	+	+	+	+	+
**ABM441**	H1	2019	ICU	-	-	-	-	-	-	ND	ND	-	-	-	-	-	ND	ND	No AbGRI2	+	-	-	-	+	-	AbGRI3$_{ABI221}$	-	ND	-	+	-	-	ND	ND	ND	ND	ND	ND
**ABM442**	H1	2019	ICU	-	-	-	-	-	-	ND	ND	-	-	-	-	-	ND	ND	No AbGRI2	+	-	-	-	+	-	AbGRI3$_{ABI221}$	-	ND	-	+	-	-	ND	ND	ND	ND	ND	ND
**ABM444**	H1	2019	ICU	-	-	-	-	-	-	ND	ND	-	-	-	-	-	ND	ND	No AbGRI2	+	-	-	-	+	-	AbGRI3$_{ABI221}$	-	ND	-	-	ND	ND	ND	ND	ND	ND	ND	ND
**ABM445**	H1	2019	ICU	-	-	-	-	-	-	ND	ND	-	-	-	-	-	ND	ND	No AbGRI2	+	-	-	-	+	-	AbGRI3$_{ABI221}$	-	ND	-	+	+	+	+	+	+	+	+	+
**ABM459**	H1	2019	Internal Ward	-	-	-	-	-	-	ND	ND	-	-	-	-	-	ND	ND	No AbGRI2	+	-	-	+	+	-	AbGRI3$_{ABI221}$	-	ND	-	-	ND	ND	ND	ND	ND	ND	ND	ND
**ABM460**	H1	2019	ICU	-	-	-	-	-	-	ND	ND	-	-	-	-	-	ND	ND	No AbGRI2	+	-	-	+	+	-	AbGRI3$_{ABI221}$	-	ND	-	+	-	-	ND	ND	ND	ND	ND	ND
**ABM461**	H1	2019	ICU	-	-	-	-	-	-	ND	ND	-	-	-	-	-	ND	ND	No AbGRI2	+	-	-	+	+	-	AbGRI3$_{ABI221}$	-	ND	-	+	-	-	ND	ND	ND	ND	ND	ND

**Isolate**	Hospital	Year	Ward	*aacA4*	*aac(6')-Im*	*aacC2*	*aacC1*	*aadA1*	*aphA1b*	IS26-*aphA1b*	*aphA1b*-IS26	AB57_1175 - *tnpR*$_1$	*bla*$_{TEM}$- *tnpA*$_{1000}$	*tnpR*$_{5393}$*c*- *aphA1b*	*aphA1*- *sul1*	*tnpA*$_{21}$-AB57_1209	TE32_13140-*tnpR1*	*aphA1b*-ABA1_01228	AbGRI2	*armA*	*atr*	*Δatr-repAciN*	*Δatr*-ISAba24	*armA-asrΔ*	*aphA1b-Δasr*	AbGRI3	*aadB*	pRAY	*intI1*	*aphA6*	ISAba125-*aph6*	*aph6*- ISAba125	Tn*aphA6*_L	Tn*aphA6*_R	*repAci6*	Repeated sequence 1	Repeated sequence 2	Repeated sequence 3
**ABM462**	H1	2019	ICU	-	-	-	-	-	-	ND	ND	-	-	-	-	-	ND	ND	No AbGRI2	+	-	-	+	+	-	AbGRI3$_{ABI221}$	-	ND	-	+	-	-	ND	ND	ND	ND	ND	ND
**ABM463**	H1	2019	Internal Ward	-	-	-	-	-	-	ND	ND	-	-	-	-	-	ND	ND	No AbGRI2	+	-	+	-	+	-	AbGRI3-4	-	ND	-	-	ND	ND	ND	ND	ND	ND	ND	ND
**ABM465**	H1	2019	Surgery	-	-	-	-	-	+	+	+	-	+	+	-	-	+	+	AbGRI2-12b	+	-	+	-	+	-	AbGRI3-4	-	ND	-	-	ND	ND	ND	ND	ND	ND	ND	ND
**ABM466**	H1	2019	Surgery	-	-	-	-	-	-	ND	ND	-	-	-	-	-	ND	ND	No AbGRI2	+	-	-	+	+	-	AbGRI3$_{ABI221}$	-	ND	-	+	+	+	-	-	ND	ND	ND	ND
**ABM467**	H1	2019	ICU	-	-	-	-	-	-	ND	ND	-	-	-	-	-	ND	ND	No AbGRI2	+	-	+	-	+	-	AbGRI3-4	-	ND	-	-	ND	ND	ND	ND	ND	ND	ND	ND
**ABM468**	H1	2019	Surgery	-	-	-	-	+	-	ND	ND	-	-	-	-	-	ND	ND	No AbGRI2	+	-	-	+	+	-	AbGRI3$_{ABI221}$	-	ND	-	+	-	+	-	-	ND	ND	ND	ND
**ABM469**	H1	2019	Internal Ward	-	-	-	-	-	-	ND	ND	-	-	-	-	-	ND	ND	No AbGRI2	+	-	-	+	+	-	AbGRI3$_{ABI221}$	-	ND	-	+	-	-	ND	ND	ND	ND	ND	ND
**ABM471**	H1	2019	Heart Surgery	-	-	-	-	-	-	ND	ND	-	-	-	-	-	ND	ND	No AbGRI2	+	-	-	+	+	-	AbGRI3$_{ABI221}$	-	ND	-	+	-	-	ND	ND	ND	ND	ND	ND
**ABM472**	H1	2019	Internal Ward	-	-	-	+	-	+	+	+	-	+	+	-	-	+	+	AbGRI2-12b	-	+	ND	ND	ND	ND	No AbGRI3	-	ND	+	+	-	-	ND	ND	ND	ND	ND	ND
**ABM473**	H1	2019	Internal Ward	-	-	-	-	-	-	ND	ND	-	-	-	-	-	ND	ND	No AbGRI2	+	-	-	-	+	-	AbGRI3$_{ABI221}$	+	+	-	-	ND	ND	ND	ND	ND	ND	ND	ND
**ABM474**	H1	2019	ICU	-	-	-	-	-	-	ND	ND	-	-	-	-	-	ND	ND	No AbGRI2	+	-	-	+	+	-	AbGRI3$_{ABI221}$	+	+	-	-	ND	ND	ND	ND	ND	ND	ND	ND
**ABM475**	H1	2019	CCU	-	-	-	-	-	-	ND	ND	-	-	-	-	-	ND	ND	No AbGRI2	+	-	+	-	+	-	AbGRI3-4	+	+	-	-	ND	ND	ND	ND	ND	ND	ND	ND
**ABM476**	H1	2019	Internal Ward	-	-	-	-	-	-	ND	ND	-	-	-	-	-	ND	ND	No AbGRI2	+	-	+	-	+	-	AbGRI3-4	+	+	-	+	-	-	ND	ND	ND	ND	ND	ND
**ABH001**	H2	2012	NR	-	-	-	-	-	-	ND	ND	-	-	-	-	-	ND	ND	No AbGRI2	+	-	+	-	+	-	AbGRI3-4	-	ND	-	+	+	+	+	+	+	+	+	+
**ABH003**	H2	2012	NR	-	-	-	-	-	-	ND	ND	-	-	-	-	-	ND	ND	No AbGRI2	+	-	+	-	+	-	AbGRI3-4	-	ND	-	+	-	-	ND	ND	ND	ND	ND	ND
**ABH006**	H2	2012	NR	-	-	-	-	-	-	ND	ND	-	-	-	-	-	ND	ND	No AbGRI2	+	-	+	-	+	-	AbGRI3-4	-	ND	-	+	+	+	+	+	+	+	+	+
**ABH007**	H2	2012	NR	-	-	-	-	-	-	ND	ND	-	-	-	-	-	ND	ND	No AbGRI2	+	-	+	-	+	-	AbGRI3-4	-	ND	-	+	+	+	+	+	+	+	+	+
**ABH013**	H2	2012	NR	-	-	-	+	-	-	ND	ND	-	-	-	-	-	ND	ND	No AbGRI2	+	-	+	-	+	-	AbGRI3-4	-	ND	-	+	+	+	+	+	+	+	+	+
**ABH014**	H2	2012	NR	-	-	-	-	-	-	ND	ND	-	-	-	-	-	ND	ND	No AbGRI2	+	-	+	-	+	-	AbGRI3-4	-	ND	-	-	ND	ND	ND	ND	ND	ND	ND	ND
**ABH019**	H2	2012	NR	-	-	-	-	-	+	+	+	-	+	+	-	-	+	+	AbGRI2-12b	+	-	+	-	+	-	AbGRI3-4	-	ND	-	-	ND	ND	ND	ND	ND	ND	ND	ND
**ABH065**	H2	2013	ICU	-	-	-	-	-	-	ND	ND	-	-	-	-	-	ND	ND	No AbGRI2	+	-	+	-	+	-	AbGRI3-4	+	+	-	+	+	+	+	+	+	+	+	+

a. Not recorded

ND1, 2. Not defined; Theses PCRs were only performed for the isolates containing the *aphA1b* gene.

ND3, 4. Not defined; Theses PCRs were only performed for the isolates containing both *bla*$_{TEM}$- *tnpA1000* and *tnpR*$_{5393c}$- *aphA1*.

ND5-8. Not defined; Theses PCRs were not performed for the isolates containing intact *atr* gene.

ND9. Not defined; This PCR was only performed for the isolates containing the *aadB* gene.

ND10-11. Not defined; Theses PCRs were only performed for the isolates containing the *aphA6* gene.

ND12-13. Not defined; Theses PCRs were only performed for the isolates containing the Tn*aphA6*

ND14-17. Not defined; Theses PCRs were only performed for the isolates that the Tn*aphA6* was on the plasmid backbone.

Table S5. Characteristics of the GC2 isolates containing AbGRI1 resistance island.

Isolate	Hospital	Year	Ward	*comM*	J1	J2	Backbone transposon						*strA* and *strB* (aminoglycoside resistance genes)							orf region			Non-aminoglycoside resistance genes						Pattern
							orf4b-*comM*	*tniBΔ-tniEΔ*	*tniB-tniE*	*tniB-tniD*	*tniD-uspA*	*comM* -Tn	*strA*	*strB*	*strA-strB*	*strA-comM*	*strB* - orf4b	CR2	**CR2- *strB***	orf6- orf7	*int*- orf11	orf9- *tniCb*	*tetA(B)*	*tetR(B)*	***tetA(B)- tetR(B)***	*tetR(B)*- CR2	*sul2*	ISAba1- *sul2*	
ABS564	H4	2019	General ICU	-	+	+	-	ND	ND	ND	ND	ND	-	-	-	-	-	-	-	ND	ND	ND	ND	ND	ND	ND	ND	ND	-
ABS567	H4	2019	General ICU	-	+	+	-	ND	ND	ND	ND	ND	-	-	-	-	-	-	-	ND	ND	ND	ND	ND	ND	ND	ND	ND	-
ABS568	H4	2019	Internal ICU	-	+	+	-	ND	ND	ND	ND	ND	-	-	-	-	-	-	-	ND	ND	ND	ND	ND	ND	ND	ND	ND	-
ABS569	H4	2019	General ICU	-	+	+	-	ND	ND	ND	ND	ND	-	-	-	-	-	-	-	ND	ND	ND	ND	ND	ND	ND	ND	ND	-
ABS570	H4	2019	General ICU	-	+	+	-	ND	ND	ND	ND	ND	-	-	-	-	-	-	-	ND	ND	ND	ND	ND	ND	ND	ND	ND	-
ABS571	H4	2019	Heart CCU	-	+	+	-	ND	ND	ND	ND	ND	-	-	-	-	-	-	-	ND	ND	ND	ND	ND	ND	ND	ND	ND	-
ABS572	H4	2019	Heart ICU	-	+	+	-	ND	ND	ND	ND	ND	-	-	-	-	-	-	-	ND	ND	ND	ND	ND	ND	ND	ND	ND	-
ABS574	H4	2019	Hematology oncology 3	-	+	+	-	ND	ND	ND	ND	ND	-	-	-	-	-	-	-	ND	ND	ND	ND	ND	ND	ND	ND	ND	-
ABS575	H4	2019	General ICU	-	+	+	-	ND	ND	ND	ND	ND	-	-	-	-	-	-	-	ND	ND	ND	ND	ND	ND	ND	ND	ND	-
ABS577	H4	2019	General ICU	-	+	+	-	ND	ND	ND	ND	ND	-	-	-	-	-	-	-	ND	ND	ND	ND	ND	ND	ND	ND	ND	-

Isolate	Hospital	Year	Ward	*comM*	J1	J2	Backbone transposon						*strA* and *strB* (aminoglycoside resistance genes)							orf region			Non-aminoglycoside resistance genes						Pattern
							orf4b-*comM*	*tniBΔ-tniEΔ*	*tniB-tniE*	*tniB-tniD*	*tniD-uspA*	*comM* -Tn	*strA*	*strB*	*strA-strB*	*strA-comM*	*strB* - orf4b	CR2	**CR2- *strB***	orf6- orf7	*int*- orf11	orf9- *tniCb*	*tetA(B)*	*tetR(B)*	***tetA(B)- tetR(B)***	*tetR(B)*- CR2	*sul2*	ISAba1- *sul2*	
ABS580	H4	2019	Surgery	-	+	+	-	ND	ND	ND	ND	ND	-	-	-	-	-	-	-	ND	ND	ND	ND	ND	ND	ND	ND	ND	-
ABS581	H4	2019	Internal ICU	-	+	+	-	ND	ND	ND	ND	ND	-	-	-	-	-	-	-	ND	ND	ND	ND	ND	ND	ND	ND	ND	-
ABS582	H4	2019	Surgery	-	+	+	-	ND	ND	ND	ND	ND	-	-	-	-	-	-	-	ND	ND	ND	ND	ND	ND	ND	ND	ND	-
ABS583	H4	2019	General ICU	-	+	+	-	ND	ND	ND	ND	ND	-	-	-	-	-	-	-	ND	ND	ND	ND	ND	ND	ND	ND	ND	-
ABS588	H5	2019	Burn 2	-	+	+	-	ND	ND	ND	ND	ND	-	-	-	-	-	-	-	ND	ND	ND	ND	ND	ND	ND	ND	ND	-
ABS593	H5	2019	Burn 2	-	+	+	-	ND	ND	ND	ND	ND	-	-	-	-	-	-	-	ND	ND	ND	ND	ND	ND	ND	ND	ND	-
ABS614	H5	2019	Burn 2	-	+	+	-	ND	ND	ND	ND	ND	-	-	-	-	-	-	-	ND	ND	ND	ND	ND	ND	ND	ND	ND	-
ABM304	H1	2018	General ICU	-	+	+	-	ND	ND	ND	ND	ND	-	-	-	-	-	-	-	ND	ND	ND	ND	ND	ND	ND	ND	ND	-
ABM305	H1	2018	General ICU	-	+	+	-	ND	ND	ND	ND	ND	-	-	-	-	-	-	-	ND	ND	ND	ND	ND	ND	ND	ND	ND	-
ABM310	H1	2018	Internal ICU	-	+	+	-	ND	ND	ND	ND	ND	-	-	-	-	-	-	-	ND	ND	ND	ND	ND	ND	ND	ND	ND	-
ABM313	H1	2019	General ICU	-	+	+	-	ND	ND	ND	ND	ND	-	-	-	-	-	-	-	ND	ND	ND	ND	ND	ND	ND	ND	ND	-

Isolate	Hospital	Year	Ward	*comM*	J1	J2	Backbone transposon						*strA* and *strB* (aminoglycoside resistance genes)							orf region			Non-aminoglycoside resistance genes						Pattern
							orf4b-*comM*	*tniBΔ-tniEΔ*	*tniB-tniE*	*tniB-tniD*	*tniD-uspA*	*comM* -Tn	*strA*	*strB*	*strA-strB*	*strA-comM*	*strB* - orf4b	CR2	**CR2- *strB***	orf6- orf7	*int*- orf11	orf9- *tniCb*	*tetA(B)*	*tetR(B)*	***tetA(B)- tetR(B)***	*tetR(B)*- CR2	*sul2*	ISAba1- *sul2*	
ABM315	H1	2019	NR	-	+	+	-	ND	ND	ND	ND	ND	-	-	-	-	-	-	-	ND	ND	ND	ND	ND	ND	ND	ND	ND	-
ABM316	H1	2019	Internal ICU	-	+	+	-	ND	ND	ND	ND	ND	-	-	-	-	-	-	-	ND	ND	ND	ND	ND	ND	ND	ND	ND	-
ABM322	H1	2019	ICU	-	+	+	-	ND	ND	ND	ND	ND	-	-	-	-	-	-	-	ND	ND	ND	ND	ND	ND	ND	ND	ND	-
ABM323	H1	2019	CCU	-	+	+	-	ND	ND	ND	ND	ND	-	-	-	-	-	-	-	ND	ND	ND	ND	ND	ND	ND	ND	ND	-
ABM324	H1	2019	Internal Ward	-	+	+	-	ND	ND	ND	ND	ND	-	-	-	-	-	-	-	ND	ND	ND	ND	ND	ND	ND	ND	ND	-
ABM331	H1	2019	ICU	-	+	+	-	ND	ND	ND	ND	ND	-	-	-	-	-	-	-	ND	ND	ND	ND	ND	ND	ND	ND	ND	-
ABM334	H1	2019	ICU	-	+	+	-	ND	ND	ND	ND	ND	-	-	-	-	-	-	-	ND	ND	ND	ND	ND	ND	ND	ND	ND	-
ABM336	H1	2019	ICU	-	+	+	-	ND	ND	ND	ND	ND	-	-	-	-	-	-	-	ND	ND	ND	ND	ND	ND	ND	ND	ND	-
ABM337	H1	2019	ICU	-	+	+	-	ND	ND	ND	ND	ND	-	-	-	-	-	-	-	ND	ND	ND	ND	ND	ND	ND	ND	ND	-
ABM341	H1	2019	Internal Ward	-	+	+	-	ND	ND	ND	ND	ND	-	-	-	-	-	-	-	ND	ND	ND	ND	ND	ND	ND	ND	ND	-
ABM342	H1	2019	Surgery	-	+	+	-	ND	ND	ND	ND	ND	-	-	-	-	-	-	-	ND	ND	ND	ND	ND	ND	ND	ND	ND	-

Isolate	Hospital	Year	Ward	*comM*	J1	J2	Backbone transposon						*strA* and *strB* (aminoglycoside resistance genes)							orf region			Non-aminoglycoside resistance genes						Pattern
							orf4b-*comM*	*tniBA-tniEΔ*	*tniB-tniE*	*tniB-tniD*	*tniD-uspA*	*comM* -Tn	*strA*	*strB*	*strA-strB*	*strA-comM*	*strB* - orf4b	CR2	**CR2- *strB***	orf6- orf7	*int*- orf11	orf9- *tniCb*	*tetA(B)*	*tetR(B)*	***tetA(B)- tetR(B)***	*tetR(B)*- CR2	*sul2*	ISAba1- *sul2*	
ABM343	H1	2019	ICU	-	+	+	-	ND	ND	ND	ND	ND	-	-	-	-	-	-	-	ND	ND	ND	ND	ND	ND	ND	ND	ND	-
ABM346	H1	2019	Internal Ward	-	+	+	-	ND	ND	ND	ND	ND	-	-	-	-	-	-	-	ND	ND	ND	ND	ND	ND	ND	ND	ND	-
ABM366	H1	2019	Internal ICU	-	+	+	-	ND	ND	ND	ND	ND	-	-	-	-	-	-	-	ND	ND	ND	ND	ND	ND	ND	ND	ND	-
ABM368	H1	2019	Internal ICU	-	+	+	-	ND	ND	ND	ND	ND	-	-	-	-	-	-	-	ND	ND	ND	ND	ND	ND	ND	ND	ND	-
ABM377	H1	2019	Internal Ward	-	+	+	-	ND	ND	ND	ND	ND	-	-	-	-	-	-	-	ND	ND	ND	ND	ND	ND	ND	ND	ND	-
ABM379	H1	2019	ICU	-	+	+	-	ND	ND	ND	ND	ND	-	-	-	-	-	-	-	ND	ND	ND	ND	ND	ND	ND	ND	ND	-
ABM380	H1	2019	ICU	-	+	+	-	ND	ND	ND	ND	ND	-	-	-	-	-	-	-	ND	ND	ND	ND	ND	ND	ND	ND	ND	-
ABM382	H1	2019	ICU	-	+	+	-	ND	ND	ND	ND	ND	-	-	-	-	-	-	-	ND	ND	ND	ND	ND	ND	ND	ND	ND	-
ABM391	H1	2019	ICU	-	+	+	-	ND	ND	ND	ND	ND	-	-	-	-	-	-	-	ND	ND	ND	ND	ND	ND	ND	ND	ND	-
ABM395	H1	2019	ICU	-	+	+	-	ND	ND	ND	ND	ND	-	-	-	-	-	-	-	ND	ND	ND	ND	ND	ND	ND	ND	ND	-
ABM399	H1	2019	ICU	-	+	+	-	ND	ND	ND	ND	ND	-	-	-	-	-	-	-	ND	ND	ND	ND	ND	ND	ND	ND	ND	-

Isolate	Hospital	Year	Ward	*comM*	J1	J2	Backbone transposon						*strA* and *strB* (aminoglycoside resistance genes)							orf region			Non-aminoglycoside resistance genes						Pattern
							orf4b-*comM*	*tniBΔ-tniEΔ*	*tniB-tniE*	*tniB-tniD*	*tniD-uspA*	*comM* -Tn	*strA*	*strB*	*strA-strB*	*strA-comM*	*strB* - orf4b	CR2	**CR2- *strB***	orf6- orf7	*int*- orf11	orf9- *tniCb*	*tetA(B)*	*tetR(B)*	***tetA(B)- tetR(B)***	*tetR(B)*- CR2	*sul2*	ISAba1- *sul2*	
ABM402	H1	2019	ICU	-	+	+	-	ND	ND	ND	ND	ND	-	-	-	-	-	-	-	ND	ND	ND	ND	ND	ND	ND	ND	ND	-
ABM429	H1	2019	ICU	-	+	+	-	ND	ND	ND	ND	ND	-	-	-	-	-	-	-	ND	ND	ND	ND	ND	ND	ND	ND	ND	-
ABM432	H1	2019	Internal ICU	-	+	+	-	ND	ND	ND	ND	ND	-	-	-	-	-	-	-	ND	ND	ND	ND	ND	ND	ND	ND	ND	-
ABM435	H1	2019	ICU	-	+	+	-	ND	ND	ND	ND	ND	-	-	-	-	-	-	-	ND	ND	ND	ND	ND	ND	ND	ND	ND	-
ABM438	H1	2019	Surgery	-	+	+	-	ND	ND	ND	ND	ND	-	-	-	-	-	-	-	ND	ND	ND	ND	ND	ND	ND	ND	ND	-
ABM444	H1	2019	ICU	-	+	+	-	ND	ND	ND	ND	ND	-	-	-	-	-	-	-	ND	ND	ND	ND	ND	ND	ND	ND	ND	-
ABM463	H1	2019	Internal Ward	-	+	+	-	ND	ND	ND	ND	ND	-	-	-	-	-	-	-	ND	ND	ND	ND	ND	ND	ND	ND	ND	-
ABM465	H1	2019	Surgery	-	+	+	-	ND	ND	ND	ND	ND	-	-	-	-	-	-	-	ND	ND	ND	ND	ND	ND	ND	ND	ND	-
ABS565	H4	2019	Internal General	-	+	+	+	+	+	+	+	+	+	+	+	+	+	+	+	+	+	+	+	+	+	+	+	+	1
ABM329	H1	2019	Outpatient	-	+	+	+	+	+	+	+	+	+	+	+	+	+	+	+	+	+	+	+	+	+	+	+	+	1
ABM338	H1	2019	ICU	-	+	+	+	+	+	+	+	+	+	+	+	+	+	+	+	+	+	+	+	+	+	+	+	+	1
ABM345	H1	2019	EmergencyB	-	+	+	+	+	+	+	+	+	+	+	+	+	+	+	+	+	+	+	+	+	+	+	+	+	1

Isolate	Hospital	Year	Ward	*comM*	J1	J2	Backbone transposon						*strA* and *strB* (aminoglycoside resistance genes)							orf region			Non-aminoglycoside resistance genes						Pattern
							orf4b-*comM*	*tniBΔ-tniEΔ*	*tniB-tniE*	*tniB-tniD*	*tniD-uspA*	*comM* -Tn	*strA*	*strB*	*strA-strB*	*strA-comM*	*strB* - orf4b	CR2	**CR2- *strB***	orf6- orf7	*int-* orf11	orf9- *tniCb*	*tetA(B)*	*tetR(B)*	***tetA(B)- tetR(B)***	*tetR(B)*- CR2	*sul2*	ISAba1- *sul2*	
ABM445	H1	2019	ICU	-	+	+	+	+	+	+	+	+	+	+	+	+	+	+	+	+	+	+	+	+	+	+	+	+	1
ABH003	H2	2012	NR	-	+	+	+	+	+	+	+	+	+	+	+	+	+	+	+	+	+	+	+	+	+	+	+	+	1
ABH006	H2	2012	NR	-	+	+	+	+	+	+	+	+	+	+	+	+	+	+	+	+	+	+	+	+	+	+	+	+	1
ABH007	H2	2012	NR	-	+	+	+	+	+	+	+	+	+	+	+	+	+	+	+	+	+	+	+	+	+	+	+	+	1
ABH013	H2	2012	NR	-	+	+	+	+	+	+	+	+	+	+	+	+	+	+	+	+	+	+	+	+	+	+	+	+	1
ABH014	H2	2012	NR	-	+	+	+	+	+	+	+	+	+	+	+	+	+	+	+	+	+	+	+	+	+	+	+	+	1
ABH019	H2	2012	NR	-	+	+	+	+	+	+	+	+	+	+	+	+	+	+	+	+	+	+	+	+	+	+	+	+	1
ABH065	H2	2013	ICU	-	+	+	+	+	+	+	+	+	+	+	+	+	+	+	+	+	+	+	+	+	+	+	+	+	1
ABS594	H5	2019	Burn 2	-	+	+	+	+	+	+	+	+	+	+	+	+	+	+	+	-	-	-	+	+	+	+	+	+	2
ABM434	H1	2019	ICU	-	+	+	+	+	+	+	+	+	+	+	+	+	+	+	+	-	-	-	+	+	+	+	+	+	2
ABM378	H1	2019	Surgery	-	+	+	+	+	+	+	+	+	+	+	+	+	+	+	+	+	+	+	+	+	+	+	-	-	3
ABM390	H1	2019	Surgery	-	+	+	+	+	+	+	+	+	+	+	+	+	+	+	+	+	+	+	+	+	+	+	-	-	3
ABS573	H4	2019	Internal ICU	-	+	+	+	+	-	-	-	+	+	+	+	+	+	+	+	+	+	+	+	+	+	+	+	+	4

Isolate	Hospital	Year	Ward	*comM*	J1	J2	Backbone transposon						*strA* and *strB* (aminoglycoside resistance genes)							orf region			Non-aminoglycoside resistance genes						Pattern
							orf4b-*comM*	*tniBΔ-tniEΔ*	*tniB-tniE*	*tniB-tniD*	*tniD-uspA*	*comM* -Tn	*strA*	*strB*	*strA-strB*	*strA-comM*	*strB* - orf4b	CR2	**CR2- *strB***	orf6- orf7	*int*- orf11	orf9- *tniCb*	*tetA(B)*	*tetR(B)*	***tetA(B)- tetR(B)***	*tetR(B)*- CR2	*sul2*	ISAba1- *sul2*	
ABM428	H1	2019	Internal ICU	-	+	+	+	+	-	-	-	+	+	+	+	+	+	+	+	+	+	+	+	+	+	+	+	+	4
ABM433	H1	2019	Internal Ward	-	+	+	+	+	-	-	-	+	+	+	+	+	+	+	+	+	+	+	+	+	+	+	+	+	4
ABM440	H1	2019	ICU	-	+	+	+	+	-	-	-	+	+	+	+	+	+	+	+	+	+	+	+	+	+	+	+	+	4
ABM442	H1	2019	ICU	-	+	+	+	+	-	-	-	+	+	+	+	+	+	+	+	+	+	+	+	+	+	+	+	+	4
ABM459	H1	2019	Internal Ward	-	+	+	+	+	-	-	-	+	+	+	+	+	+	+	+	+	+	+	+	+	+	+	+	+	4
ABM466	H1	2019	Surgery	-	+	+	+	+	-	-	-	+	+	+	+	+	+	+	+	+	+	+	+	+	+	+	+	+	4
ABM472	H1	2019	Internal Ward	-	+	+	+	+	-	-	-	+	+	+	+	+	+	+	+	+	+	+	+	+	+	+	+	+	4
ABM473	H1	2019	Internal Ward	-	+	+	+	+	-	-	-	+	+	+	+	+	+	+	+	+	+	+	+	+	+	+	+	+	4
ABM474	H1	2019	ICU	-	+	+	+	+	-	-	-	+	+	+	+	+	+	+	+	+	+	+	+	+	+	+	+	+	4
ABM475	H1	2019	CCU	-	+	+	+	+	-	-	-	+	+	+	+	+	+	+	+	-	-	-	+	+	+	+	+	+	4
ABM476	H1	2019	Internal Ward	-	+	+	+	+	-	-	-	+	+	+	+	+	+	+	+	+	+	+	+	+	+	+	+	+	4
ABH001	H2	2012	NR	-	+	+	+	+	-	-	-	+	+	+	+	+	+	+	+	+	+	+	+	+	+	+	+	+	4

Isolate	Hospital	Year	Ward	*comM*	J1	J2	Backbone transposon						*strA* and *strB* (aminoglycoside resistance genes)							orf region			Non-aminoglycoside resistance genes						Pattern
							orf4b-*comM*	*tniBΔ-tniEΔ*	*tniB-tniE*	*tniB-tniD*	*tniD-uspA*	*comM* -Tn	*strA*	*strB*	*strA-strB*	*strA-comM*	*strB* - orf4b	CR2	**CR2- *strB***	orf6- orf7	*int*- orf11	orf9- *tniCb*	*tetA(B)*	*tetR(B)*	***tetA(B)- tetR(B)***	*tetR(B)*- CR2	*sul2*	ISAba1- *sul2*	
ABS470	H4	2018	NR	-	+	+	+	+	-	-	-	+	+	+	+	+	+	+	+	+	+	+	-	-	ND	ND	+	+	5
ABS495	H4	2018	General Surgery	-	+	+	+	+	-	-	-	+	+	+	+	+	+	+	+	+	+	+	-	-	ND	ND	+	+	5
ABS496	H4	2018	General ICU	-	+	+	+	+	-	-	-	+	+	+	+	+	+	+	+	+	+	+	-	-	ND	ND	+	+	5
ABS566	H4	2019	Heart ICU	-	+	+	+	+	-	-	-	+	+	+	+	+	+	+	+	+	+	+	-	-	ND	ND	+	+	5
ABM319	H1	2019	Orthopedics	-	+	+	+	+	-	-	-	+	+	+	+	+	+	+	+	+	+	+	-	-	ND	ND	+	+	5
ABM392	H1	2019	Emergency	-	+	+	+	+	-	-	-	+	+	+	+	+	+	+	+	+	+	+	-	-	ND	ND	+	+	5
ABM393	H1	2019	Internal Ward	-	+	+	+	+	-	-	-	+	+	+	+	+	+	+	+	+	+	+	-	-	ND	ND	+	+	5
ABS534	H4	2019	Neurology ICU	-	+	+	+	+	-	-	-	+	+	+	+	+	+	+	+	-	-	-	+	+	+	+	+	+	6
ABM441	H1	2019	ICU	-	+	+	+	+	-	-	-	+	+	+	+	+	+	+	+	-	-	-	+	+	+	+	+	+	6
ABM460	H1	2019	ICU	-	+	+	+	+	-	-	-	+	+	+	+	+	+	+	+	-	-	-	+	+	+	+	+	+	6
ABM461	H1	2019	ICU	-	+	+	+	+	-	-	-	+	+	+	+	+	+	+	+	-	-	-	+	+	+	+	+	+	6
ABM462	H1	2019	ICU	-	+	+	+	+	-	-	-	+	+	+	+	+	+	+	+	-	-	-	+	+	+	+	+	+	6

Isolate	Hospital	Year	Ward	*comM*	J1	J2	Backbone transposon						*strA* and *strB* (aminoglycoside resistance genes)							orf region			Non-aminoglycoside resistance genes						Pattern
							orf4b-*comM*	*tniBΔ-tniEΔ*	*tniB-tniE*	*tniB-tniD*	*tniD-uspA*	*comM* -Tn	*strA*	*strB*	*strA-strB*	*strA-comM*	*strB* - orf4b	CR2	**CR2- *strB***	orf6- orf7	*int*- orf11	orf9- *tniCb*	*tetA(B)*	*tetR(B)*	***tetA(B)- tetR(B)***	*tetR(B)*- CR2	*sul2*	ISAba1- *sul2*	
ABM469	H1	2019	Internal Ward	-	+	+	+	+	-	-	-	+	+	+	+	+	+	+	+	-	-	-	+	+	+	+	+	+	6
ABM430	H1	2019	ICU	-	+	+	+	+	-	-	-	+	+	+	+	+	+	+	+	-	-	-	+	+	+	+	+	+	6
ABM471	H1	2019	Heart Surgery	-	+	+	+	+	-	-	-	+	+	+	+	+	+	+	+	-	-	-	+	+	+	+	-	-	7
ABM467	H1	2019	ICU	-	+	+	+	+	-	-	-	+	+	+	+	+	+	+	+	-	-	-	+	+	+	+	-	-	7
ABM468	H1	2019	Surgery	-	+	+	+	+	-	-	-	+	+	+	+	+	+	+	+	-	-	-	+	+	+	+	-	-	7

Rows highlighted in the same colour indicate the same pattern.
ND, Not determined, for the isolates without the orf4b-*comM* fragment, other PCRs were not performed.
The PCRs in bold are the linkage PCRs that were performed for identification of AbGRI1s in this study.
NR, Not recorded.
